# Precision is not limited by the second law of thermodynamics

**DOI:** 10.1038/s41567-025-02929-2

**Published:** 2025-06-02

**Authors:** Florian Meier, Yuri Minoguchi, Simon Sundelin, Tony J. G. Apollaro, Paul Erker, Simone Gasparinetti, Marcus Huber

**Affiliations:** 1 Atominstitut, Technische Universität Wien, Vienna, Austria; 2 Institute for Quantum Optics and Quantum Information – IQOQI Vienna, Austrian Academy of Sciences, Vienna, Austria; 3 Department of Microtechnology and Nanoscience, Chalmers University of Technology, Gothenburg, Sweden; 4 Department of Physics, University of Malta, Msida, Malta

**Keywords:** Single photons and quantum effects, Quantum information

## Abstract

Physical devices operating out of equilibrium are affected by thermal fluctuations, limiting their operational precision. This issue is particularly pronounced at microscopic and quantum scales, where its mitigation requires additional entropy dissipation. Understanding this constraint is important for both fundamental physics and technological design. Clocks, for example, need a thermodynamic flux towards equilibrium to measure time, resulting in a minimum entropy dissipation per clock tick. Although classical and quantum models often show a linear relationship between precision and dissipation, the ultimate bounds on this relationship remain unclear. Here we present an autonomous quantum many-body clock model that achieves clock precision that scales exponentially with entropy dissipation. This is enabled by coherent transport in a spin chain with tailored couplings, where dissipation is confined to a single link. The result demonstrates that coherent quantum dynamics can surpass the traditional thermodynamic precision limits, potentially guiding the development of future high-precision, low-dissipation quantum devices.

## Abstract (web summary)

Clock precision is thought to be fundamentally limited by entropy production in out-of-equilibrium systems. A theoretical work now introduces a quantum clock design where precision grows exponentially with dissipation.

## Main

The basic dynamical equations of physics all seem to be invariant under time reversal, that is, symmetric with respect to time. As systems become more complex, this symmetry is observed to break down. This statistical breaking of time-reversal symmetry through the second law of thermodynamics is fully compatible with reversible microphysics and seems to be the only contender for an explanation for the emergence of a clear notion of past and future in physics. The implications of this profound insight for the nature of time have long been the centre of discussion in the foundation of physics. Borrowing a dictum from Einstein, “time is what a clock measures”, clocks are the witnesses of the macroscopic breaking of reversibility. As irreversible out-of-equilibrium systems, clocks come at a fundamental thermodynamic cost—entropy dissipation^1^. In the quest for the most accurate clocks, currently based on atomic^2–4^ or possibly nuclear transitions in the future^5^, these costs are not the most pressing concern. But the quest for small, self-contained quantum control^6–11^ shifts the question about the exact relationship between dissipation and precision from a foundational one to a potentially practical one. The notion of autonomous clocks not requiring external control to run allows us to explore the ultimate dissipation limits of clocks^12–16^ and may also inform practical designs for self-contained quantum control^11^.

To quantify these limits, one has to resort to microscopic models for the clock. In such models, all the resources that the clock requires to run are explicitly accounted for within the model. Such tiniest conceivable clocks measure time by counting elementary stochastic events as ticks in a regime far away from where state-of-the-art clocks work. The clock precision can be understood as the average number of times ${{\mathcal{N}}}$ said clock ticks until it goes wrong by one tick compared with a perfect clock^12^, while the corresponding thermodynamic cost is quantified by the entropy *Σ*_tick_ dissipated per unit tick. For fixed *Σ*_tick_, one may ask what is the maximum possible clock precision. This question relates the fundamental limit of clock performance to the second law of thermodynamics. A similar fluctuation tradeoff is encountered in stochastic thermodynamics with the thermodynamic uncertainty relations (TUR). For classical stochastic systems, these limits are dictated by entropy production. Classical TUR have received considerable attention^17–20^, opening the question whether the same limits apply in the quantum domain^21–32^. For fully dissipative clocks, a linear bound ${{\mathcal{N}}}\le {\varSigma }_{{{\rm{tick}}}}/2$ tightly bounds the clock precision. Such a bound has been confirmed both theoretically^12,13^ and experimentally^33^. In certain quantum scenarios, the linear bound can be beaten by using quantum coherence beyond the dissipative regime. So far, however, only small theoretical violations have been reported^15,21–23,30^, and larger ones remain contested^24,29,34^.

**Fig. 1:**
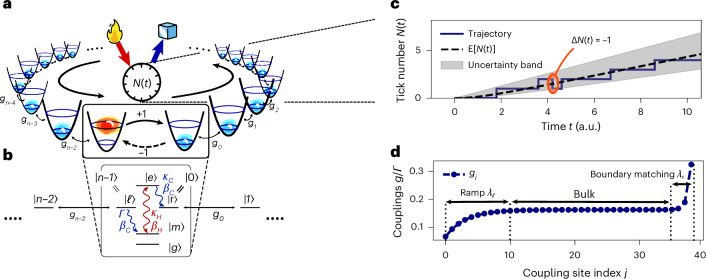
The ring clock. **a**, Schematic depiction. The clock consists of a ring of *n* quantum systems (egg cups) hosting a single excitation that travels around the ring. Upon completing one cycle, the clock ticks by undergoing a biased jump from the last to the first site. Credit: fire and icecube icons in **a**, OpenMoji under a Creative Commons license CC-BY-SA 4.0. **b**, A level diagram of a quantum system providing a directional interface between the first site and the last site of the ring using a thermal gradient. The level diagram is in the single-excitation subspace, that is, if one of the sites is in an excited state, all others are in the ground state. See the Methods for details. **c**, A representative trajectory of the number of ticks *N*(*t*) counted by the clock as a function of time (solid line). Due to thermal fluctuations, such a counter can jump backwards (highlighted jump). For the clock to be precise, such backwards jumps must be suppressed, using a strong thermal gradient. **d**, Numerically optimized couplings, *g*_*j*_, between the nearest-neighbour sites of the ring clock, for a ring of *n* = 40 sites. Based on the dependence of the coupling coefficients on the site position in the ring, well approximated by equation (6), we identify three regions. In the initial ramp region of length *λ*_*ℓ*_, an excitation present in the first site is autonomously shaped into a travelling wave packet. The bulk propagation region is akin to a delay line. Finally, the boundary matching region, of length *λ*_r_, ensures that the wave packet is absorbed from the last site without reflection.

Here, we report the discovery of a fully autonomous quantum clock model whose precision grows with entropy production as

1 $${{\mathcal{N}}}={{\mathrm{e}}}^{\varOmega ({\varSigma }_{{{\rm{tick}}}})}\,,$$

showing that dissipation does not limit clock precision. The *Ω*-notation denotes an asymptotic lower bound, ignoring constant factors^35^. The proposed quantum clock is based on a spin chain with site-dependent nearest-neighbour couplings and is stable under small perturbations of the couplings and losses along the chain as detailed in the Methods. The set-up could be realized extensibly, for example, in the circuit quantum electrodynamics architecture with coupled cavity arrays (CCAs)^36,37^, and the set-up as sketched in Fig. 1a,b. The clock works by topologically closing the spin chain to a ring and transporting a single excitation around the ring. A chirality is introduced by applying a thermal bias between the first site and the last site. This set-up, which we refer to as the ring clock, counts the net number of completed cycles as ticks (Fig. 1c).

We obtain the exponential scaling by numerically optimizing the coupling coefficients between the sites in the ring. The insight enabling this scaling is the dissipation-free coherent transport in the bulk of the ring: by adding more sites, the clock’s precision can be arbitrarily increased while the dissipation occurs only between the two sites closing the ring and therefore does not grow with the ring size. We also provide a quantitative interpretation of the obtained coefficients in terms of wave-packet reshaping and boundary matching, highlighting a connection between our model and previous techniques from optimal coherent quantum transport^38,39^, dissipative quantum transport in condensed matter^40,41^, and photonics^42,43^.

**Fig. 2:**
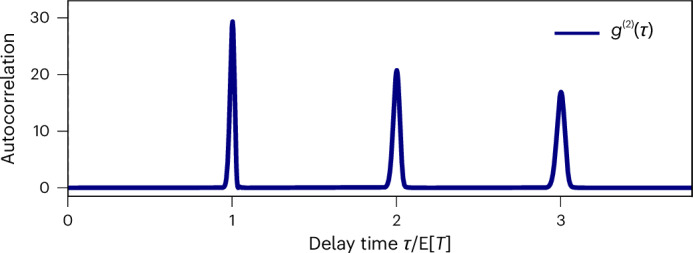
Autocorrelation function. We show *g*^(2)^(*τ*) = E[*I*(*τ*)*I*(0)]/E[*I*(0)]^2^, the autocorrelation function of the emitted ‘tick photons’ with current operator *I* = *J*^†^*J* versus delay time *τ* in the steady-state limit (details in Supplementary Section A). The graph is calculated for 350 spins, and *τ* is expressed in units of the expected time between ticks E[*T*]. By construction of the single-excitation subspace, we have anti-bunching *g*^(2)^(0) = 0, and for *τ* > 0 the deterministic, periodic emission of single photons. The peak broadening comes from accumulation of uncertainty after multiple ticks.

While our findings resolve the foundational question about whether the second law imposes a restriction on clock precision, it also holds promise beyond fundamental science. For one, a major challenge in quantum communication is that many quantum network components rely on deterministic single-photon sources^44^. State-of-the-art attempts to overcome this challenge are often based on quantum dots and require active driving^45–47^. Our result provides a self-contained model that could serve as a passive, deterministic single-photon source by heralding the regular photons emitted as the clock’s ticks (Fig. 2). For another, in distributed quantum computing, the dynamical control needed to shape photons to match the receiver and ensure the wave packet arrives precisely on time is a grand technological challenge^48,49^ that has so far been addressed with time-dependent control^50–53^. The ring clock’s design provides a pathway towards passive photon shaping using the preparation ramp of the transmission line.

## Theoretical description

The model we work with is based in the single-excitation regime of a spin chain. Thus, the relevant basis states can be written as $\left\vert 0\right\rangle := \left\vert 10\cdots 0\right\rangle$ for the state where the excitation is on the first site, $\left\vert 1\right\rangle := \left\vert 01\cdots 0\right\rangle$ for the state where it is in the second site and so on until $\left\vert n-1\right\rangle := \left\vert 0\cdots 01\right\rangle ,$ as visualized in Fig. 1a. With coherent nearest-neighbour hopping interactions, we obtain the single-excitation subspace Hamiltonian

2 $$H=\mathop{\sum }\limits_{j = 0}^{n-2}{g}_{j}\left\vert\,j\right\rangle\,\left\langle\,j+1\right\vert +{{\rm{h.c.}}},$$

where h.c. stands for the Hermitian conjugate. The constant *g*_*j*_ is a real parameter describing the coherent hopping rate between site *j* and *j* + 1. In this form, the chain of sites does not yet topologically form the desired ring. A dissipative coupling between the first site $\left\vert 0\right\rangle$ and the last site $\left\vert n-1\right\rangle$ closes the loop and is described by a Lindblad jump operator of the form $J=\sqrt{\varGamma }\left\vert 0\right\rangle \left\langle n-1\right\vert$ to model the jump in the direction $\left\vert n-1\right\rangle \to \left\vert 0\right\rangle$. In Fig. 1b, we propose a level scheme that allows such a jump process between the last and first ring site (see additional details in the Methods).

At finite entropy production, local detailed balance predicts that each jump process is accompanied by its time-reverse that is suppressed by how much entropy is produced in each jump. Here, this is described by the process $\overline{J}={{\mathrm{e}}}^{-{\varSigma }_{{{\rm{tick}}}}/2}{J}^{{\dagger} }$, whose rate is suppressed by the factor ${{\mathrm{e}}}^{-{\varSigma }_{{{\rm{tick}}}}}$ from detailed balance, and *Σ*_tick_ is the entropy produced by each unit population that undergoes the forward transition $\left\vert n-1\right\rangle \to \left\vert 0\right\rangle$. How *Σ*_tick_ relates to thermodynamic notions of heat and temperature depends on the specific realization of the biased tick transition. For example, for a clock described by the level scheme of Fig. 1b, the entropy is related to the exchanged heat *Q*, given by the energy gap between $\left\vert e\right\rangle$ and $\left\vert m\right\rangle$, and to the difference in inverse temperature *β*_*C*_, *β*_*H*_ of the environments driving the transition, according to the expression

3 $${\varSigma }_{{{\rm{tick}}}}=(\,{\beta }_{{{C}}}-{\beta }_{{{H}}})Q.$$

Given the initial state $$\rho (0)=\left\vert 0\right\rangle \left\langle 0\right\vert ,$$ we describe the evolution of the system using a quantum master equation $$\dot{\rho }=-i[H,\rho ]+{{\mathcal{D}}}[\,J]\rho +{{\mathcal{D}}}[\,\overline{J}]\rho$$ in units of *ℏ* = 1. Moreover, the dissipator is defined as $${{\mathcal{D}}}[\,J]\rho$$$$=J\rho {J}^{{\dagger} }-\frac{1}{2}\{\,{J}^{{\dagger} }J,\rho \}$$, with anticommutator {*A*, *B*} = *AB* + *BA*.

The jumps generated by *J* are counted as positive ticks and the reverse $\overline{J}$ as ‘negative ticks’, giving the number of ticks *N*(*t*) as the net completed clock cycles to estimate parameter time *t*. Clock precision can be quantified using the inverse Fano factor^16^

4 $${{{\mathcal{N}}}}_{\varSigma }=\mathop{\lim }_{t\to \infty }\frac{{{\rm{E}}}[N(t)]}{{{\rm{Var}}}[N(t)]},$$

comparing expectation value E[*N*(*t*)] with the fluctuations Var[*N*(*t*)]. For high precision, fluctuations should ideally be minimal compared with the expected number of ticks. The majority of ticks must therefore be positive, meaning the forward jump *J* should dominate over the backwards one $\overline{J}$, which requires, a priori, a high entropy production per tick *Σ*_tick_.

The goal we aim for, however, is the maximization of ${{{\mathcal{N}}}}_{\varSigma }$ by varying the Hamiltonian *H* while at the same time minimizing the entropy production per tick *Σ*_tick_. To solve this problem, we first work in the regime without the negative ticks, $\overline{J}\to 0$, requiring divergent entropy production *Σ*_tick_ → *∞*, so that only precision has to be maximized without having to handle *Σ*_tick_. As we find out later, the infinite entropy production is not needed to maintain the high precision, and the results actually hold even when the entropy production is negligibly smaller than the clock precision.

Working without negative ticks simplifies the problem of maximizing precision by making it equivalent to the problem of minimizing the relative variance of the waiting time *T* between ticks. The reason for this is that, because the clock resets to the same state $\left\vert 0\right\rangle$ after every tick, *N*(*t*) can be mapped to a renewal process^16,54^ and, thus, the central limit applies (see details in the Methods and Supplementary Section D1), giving

5 $${{{\mathcal{N}}}}_{\varSigma }\to {{{\mathcal{N}}}}_{\infty }=\frac{{{\rm{E}}}{[T\,]}^{2}}{{{\rm{Var}}}[T\,]},$$

where E[*T*] is the expected time between two ticks and Var[*T*] is the variance. This precision ${{{\mathcal{N}}}}_{\infty }$ defined relative to *T* is what has been traditionally considered in the field of quantum clocks as the main figure of merit^12,15,16,55–57^.

Working in the waiting time picture, we can maximize the clock precision by maximizing ${{{\mathcal{N}}}}_{\infty }$ as defined in equation (5). Because clock precision is timescale invariant, we can fix without loss of generality the jump rate ${\varGamma}$ and determine the coupling constants *g*_*j*_ of the Hamiltonian *H* that maximize ${{{\mathcal{N}}}}_{\infty }$. A global numerical maximization yields coupling constants *g*_*j*_ that split the ring into three regions as shown in Fig. 1d. Physically, the three regions are responsible for

1. a wave-packet preparation ramp with increasing couplings on a length scale *λ*_*ℓ*_;
2. a propagation region in the middle of the ring with flat couplings;
3. an emission region at the end of the ring apodized on a length scale *λ*_*ℓ*_, to prevent reflection.

**Extended Data Fig. 1:**
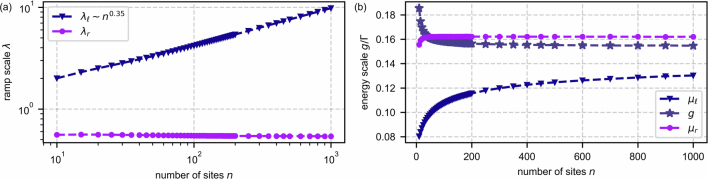
Optimized couplings. Plot of the numerically optimized parameters *μ*_*ℓ*_, *μ*_*r*_, *g*, *λ*_*ℓ*_, *λ*_*r*_ as a function of the ring length *n*. In (**a**), we show the ramp lengths *λ*_*ℓ*_ and *λ*_*r*_, finding that the left ramp grows as *n*^0.35^ close to the predicted exponent of 1/3. The exponent is determined with linear regression and has uncertainty of order 10^−6^ and is thus not shown in the figure. The right ramp does not become larger with the ring length, indicating an agreement with previous literature on apodization showing that only a constant number of couplings have to modified in a coupled cavity array to avoid reflection at a dissipative sink [42]. In (**b**), we show how *μ*_*ℓ*_, *μ*_*r*_ and *g* change as a function of *n* showing that both *g* and *μ*_*r*_ quickly approach a constant value with growing *n*, whereas *μ*_*ℓ*_ only slowly increases, indicating that the depth of the left ramp keeps increasing with *n*.

We find that the site dependence of the coupling coefficient is well approximated by

6 $${g}_{j}=-{\mu }_{\ell }{{\mathrm{e}}}^{-j/{\lambda }_{\ell }}+g+{\mu }_{r}{{\mathrm{e}}}^{\left(j-(n-1)\right)/{\lambda }_{r}},$$

where *μ*_*ℓ*_, *g* and *μ*_*r*_ are variable coupling parameters and *λ*_*ℓ*_ and *λ*_*r*_ are the length scale of the exponential ramps (see numerically optimized parameters in Extended Data Fig. 1).

Region (1) autonomously shapes the initially localized excitation at $\left\vert 0\right\rangle$ into a travelling wave packet. This problem is of independent interest^48,49^ and has usually been addressed with actively controlled, time-dependent nearest-neighbour couplings^45,52,58^, and here we solve it with passive, time-independent couplings. In the limit of large times and displacement 2*gt*, *x* ≫ *λ*_*ℓ*_ when the wave packet has already left the ramp, its width is determined by the ramp length and scales with *λ*_*ℓ*_. The scaling form can be derived in a hydrodynamical continuum limit for long ramps and large number of sites *λ*_*ℓ*_, *n* ≫ 1 (Methods). In the bulk (2), the Hamiltonian *H* can be approximated by a constant coupling, isotropic *XY* spin chain Hamiltonian^59^. This model is analytically diagonalizable with the dispersion relation $E(k)=2g\cos (k)$ and discrete momenta *k* ∈ [0, 2π) in the case of periodic boundary conditions. We find, as illustrated in Supplementary Fig. 2, that the momentum distribution of the wave packet is centred around *k*_0_ = π/2, the point where the dispersion relation is linear, and thus, the wave packet propagates nearly dispersion free. The length scale *λ*_*ℓ*_ in real space translates into the inverse length scale 1/*λ*_*ℓ*_ in momentum space, meaning that the longer the initial ramp, the more the momentum is concentrated around *k*_0_. The final region (3) is carefully matched to the decay strength *Γ* of the last site to ensure the emission of the incoming wave packet with unit probability. In our setting, the optimal couplings increase towards the emitter as shown in Fig. 1d, which is reminiscent of the couplings used to minimize reflection in resonant tunnling structures, a problem known as apodization (an expanded discussion can be found in Supplementary Section C3 and ref. ^42^).

The time between two ticks is determined by the tick probability density function (PDF) $$p(t)=\varGamma | \langle n-1\left\vert {{\mathrm{e}}}^{-i{H}_{{{\rm{eff}}}}t}\right\vert 0\rangle {| }^{2}$$, where $${H}_{{{\rm{eff}}}}$$$$=H-i\frac{1}{2}{J}^{{\dagger} }J$$ is the effective Hamiltonian of the system including the decay back action from the jump operator *J* (see details in Supplementary Section B1). The tick PDF can be split into two contributions *p*(*t*) = *p*_0_(*t*) + *p*_1_(*t*), where *p*_0_(*t*) is the free theory without loss term *J*^†^*J* and without the right ramp, and *p*_1_(*t*) is the interaction part to restore the equality with the tick PDF. While the ineraction part can formally be obtained as a Dyson series, the free theory is given by the overlap of the wave function with $$\left\vert n-1\right\rangle$$ at time *t* (see details in Supplementary Section C3). To good approximation, *p*_0_(*t*) dominates the tick time, that is, E[*T*] ~ *n*/(2*g*) is the time the excitation takes to arrive at the final site with velocity 2*g* at the quantum speed limit^60^. Similarly, the uncertainty of the arrival time $${{\rm{Var}}}[T\,] \sim {\lambda }_{\ell }^{2}/{(2g)}^{2}$$ is given by the wave packet’s width *λ*_*ℓ*_, where we use the symbol ‘~’ to denote asymptotic proportionality.

## Optimization results

Before discussing the numerical optimization, we analyse the precision scaling predicted by the free theory. We find that ${{{\mathcal{N}}}}_{\infty } \sim {n}^{2}/{\lambda }_{\ell }^{2}$ as long as *n* and *λ*_*ℓ*_ are sufficiently large. Although a wave packet with small width *λ*_*ℓ*_ would render the precision sufficiently large, such a packet will be delocalized in momentum space on a scale $\sim {\lambda }_{\ell }^{-1}$, leading to strong braodening around *k*_0_ = π/2 due to dispersion. These adverse effects are balanced for the choice *λ*_*ℓ*_ ~ *n*^1/3^ (see Supplementary Section C3b for details), for which we obtain the scaling ${{{\mathcal{N}}}}_{\infty } \sim {n}^{4/3}$.

**Fig. 3:**
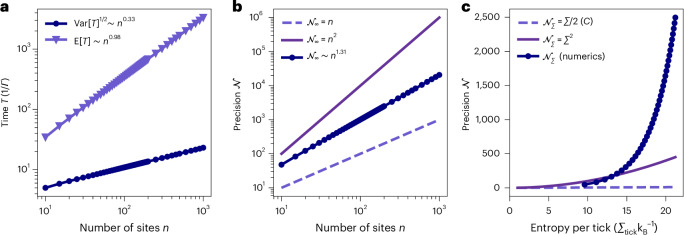
Clock performance versus ring length, *n*, and entropy production per tick, *Σ*_tick_. **a**, We show how the expected tick time E[*T*] and the (square root) variance Var[*T*]^1/2^ scale with the number of sites for the numerically optimized choice of couplings. The simulation results are in agreement with the prediction that E[*T*]$\overset{{\rm{th.}}}{\sim}$*n*^1^ and Var[*T*]$\overset{{\rm{th.}}}{\sim}$*n*^2/3^.Numerical values for the exponents are determined by linear regression, and the uncertainty in the exponent is of the order 10^−4^ and, thus, not shown in the figure. **b**, We show the clock precision ${\mathcal{N}}_{\infty }$ as a function of ring length *n* in the fully irreversible case (filled circles). For comparison, we show the classical and quantum bounds that limit precision by the dimension. The precision bound for classical stochastic clocks (dashed line) is linear in the dimension ${\mathcal{N}}_{\infty }\le n$ (ref. ^56^), whereas the one for quantum clocks (full line) scales quadratically ${\mathcal{N}}_{\infty }\le O(n^2)$ (refs. ^56,72^). **c**, We visualize the main result of this work showing that clock precision grows exponentially faster than entropy production ${\mathcal{N}}_{\varSigma }={\mathrm{e}}^{\varOmega (\varSigma _{\rm{tick}})}$ (filled circles). We compare this with the TUR bound ${\mathcal{N}}_{\varSigma }\le {\varSigma }_{\rm{tick}}/2$ that holds for classical dissipative systems (dashed lines). For further comparison, the quadratic scaling ${\mathcal{N}}_{\varSigma } \sim {\varSigma }_{\rm{tick}}^{2}$ as recently found^15^ is shown, ignoring subleading terms and constant factors (full line).

This scaling is confirmed by numerically searching for the values of *g*_*j*_ that maximize ${{{\mathcal{N}}}}_{\infty }$. We find that ${\lambda }_{\ell }^{\,{\mathrm{num.}}\,} \sim {n}^{0.35}$, for up to *n* = 1,000 sites (Extended Data Fig. 1), beyond which the computational runtime limited the simulation. Furthermore the numerics also verify the relationship between *n* and the tick time statistics, with the scaling laws E[*T*] ~ *n*^0.99^ and Var[*T*] ~ *n*^0.66^, as shown in Fig. 3a. Combined, we find that clock precision in the theory model and the numerical simulation scales as

7 $${{{\mathcal{N}}}}_{\infty } \overset{\rm{num.}}{\sim} {n}^{1.31} \overset{{\rm{th.}}}{\sim} {n}^{4/3}.$$

Up to deviations due to differences of the true and numerical maximum, and finite *n* effects, simulation (num.) and theory (th.) are in close agreement with each other (Fig. 3b).

To obtain the precision scaling (equation (7)), divergent entropy production per tick was assumed. Relaxing this idealization by going to a regime of finite entropy production *Σ*_tick_, negative ticks enter as a perturbation in the evolution equations, with perturbation parameter $\delta ={{\mathrm{e}}}^{-{\varSigma }_{{{\rm{tick}}}}}$. Large entropy per tick *Σ*_tick_ ensures a small parameter *δ* and, thus, a minimal detrimental impact on the clock precision. At the same time, a small entropy production is desirable from a resource theoretic point of view, where we wish to keep the dissipation as small as possible. A logarithmic growth of the entropy production ${\Sigma }_{{{\rm{tick}}}} \sim \log n$ with the number of sites makes the difference $| {{{\mathcal{N}}}}_{\varSigma }-{{{\mathcal{N}}}}_{\infty }|$ vanish in the limit of large *n* as we confirm numerically for up to *n* = 200 sites (see details on the theory in the Methods). This ensures that, even at finite entropy, the precision still grows polynomially in the number of sites, ${{{\mathcal{N}}}}_{\varSigma } \sim {n}^{1.31}$, giving the main result,

1 rev. $${{{\mathcal{N}}}}_{\varSigma }={{\mathrm{e}}}^{\varOmega ({\varSigma }_{{{\rm{tick}}}})},$$

that the clock precision is exponentially separated from the entropy production, as also shown in Fig. 3c.

## Outlook

In fully dissipative systems, the TUR^13,17^ have suggested that clock precision fundamentally comes at an entropic cost due to the second law, which also generalized to some quantum models^12,15^. We have developed a model system fully compatible with autonomous quantum evolution where this limitation breaks down, and in that sense, precision is not not limited by the second law of thermodynamics. The autonomy is guaranteed by combining non-equilibrium dissipative dynamics with coherent quantum evolution in a spin chain. Contrarily to other proposals of autonomous quantum clocks that increase their precision by increasing the maximum energy in the system^12,14,15,33,55^ and thereby also increase the entropy production, the ring clock works in an energy-degenerate system and through careful design of the interactions manages to exponentially improve the precision versus entropy scaling compared with the previous results.

**Extended Data Fig. 2:**
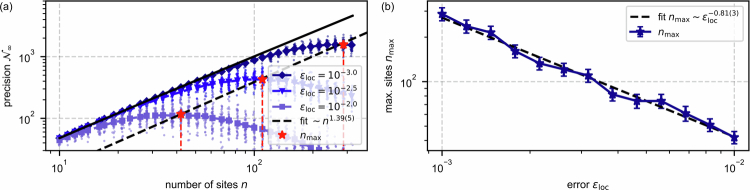
Coupling disorder. We show the precision ${{{\mathcal{N}}}}_{\infty }$ under perturbations of the couplings. (**a**) Here, the precision is shown as a function of the number of sites for a selection of three relative errors *ε*_loc_ in the parameters. The dots correspond to the instances of the clock subject to random error and the dashed line with the cross markers is the numerical average over *M* = 400 samples for fixed *ε*_loc_ and *n*. The solid black line is the optimal scaling and the red star markers are the maxima ${n}_{\max }$ of the average curve for fixed error. The dashed black line is a linear fit through all maxima and the uncertainty in the exponent is due to the statistical errors in obtaining the average. (**b**) Here, ${n}_{\max }$ as identified from the *M* = 400 samples in (**a**) is plotted as a function of the error *ε*_loc_. The error bars for ${n}_{\max }$ show the finite resolution in the number of sites *n* sampled in (**a**) as the estimated uncertainty. The linear fit of the curve determines the exponent *κ*_loc_, with the error coming from the statistical distribution of the data points.

**Extended Data Fig. 3:**
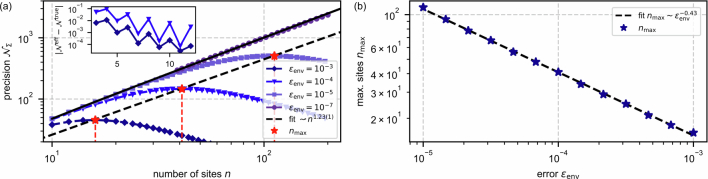
Amplitude damping noise. Precision under amplitude damping noise with strength *Γε*_env_. (**a**) Filled circles show the precision for 4 values of *ε*_env_ as calculated with the effective model. Data points where the numerical calculation was unstable have been removed. The solid black line shows the ideal precision as a function of the number of sites *n*. The red star markers correspond to the maxima ${n}_{\max }$ of each curve showing the turn-around point for fixed loss *ε* where the precision decreases with more sites, and the black dashed line is a linear fit through the maxima. Inset: differences between the precision predicted by the effective model and the full model *vs* ring size. Because of coherent effects in the double-excitation subspace not captured by the effective model, the difference vanishes at different rates for odd and even site numbers, but is negligible for all *n* ≳ 10. (**b**) This plot shows ${n}_{\max }$ as a function of the error *ε*_env_ in a double logarithmic scale, together with a linear fit.

The idealized setting of our analysis raises the question about stability: how do imperfections impact the performance? So far, we have assumed fine-tuned couplings and the absence of dissipation in the channel along the entire chain, except for the last site. In realistic settings, the couplings would not perfectly match the ideal ones, *g*_*j*_. In addition, each site would couple to a finite-temperature environment. This may lead to erroneous losses as well as absorption of excitations in the bulk of the ring. In the presence of such noise, the ring may carry more than one excitation in the clock, affecting the regularity of the ticks (see the Methods for details). Any finite perturbation of the ideal scenario thus leads to a maximum length in which the exponential scaling can still be upheld, before breaking down again, as we detail in Extended Data Figs. 2 and 3. We find that the error only needs to decrease polynomially (rather than exponentially) in the maximum length we want to achieve, showing that the exponential entropy precision relationship (equation (1)) relies neither on an exponentially precise preparation of the set-up nor on perfect isolation from the environment, thus corroborating the stability of the ring clock.

Despite these technical challenges, CCAs, for example, have been used to realize microwave metamaterials with tailored band structures^43,58,61^, also serving as slow-light waveguides^62^. They consist of an array of capacitively coupled lumped-element superconducting microwave resonators, which can exhibit low intrinsic dissipation (internal quality factors of *Q*_*i*_ ~ 10^5^), supporting arrays of up to *n* = 100 individual sites^37^. The couplings between adjacent sites are determined by the capacitive network of the circuit and can be engineered to satisfy the prescription in Fig. 1d without the need for active control. The tick-generating element of the clock (Fig. 1b) could be achieved using a superconducting artificial molecule comprising one or more artificial atoms. Through careful design of the band structure of the CCA and transition frequencies of the energy levels, selective emission from certain transitions can be achieved^62^ ensuring that only a single excitation is propagating through the CCA. Further dissipation into the environment for other transitions can be made possible through tailored dissipation engineering between the artificial atoms and microwave waveguides^63,64^ to model the respective thermal baths^65^. To detect the ticks of the clock, we envisage two methods. One method uses continuous, dispersive readout of a particular eigenstate of the molecule, marking a tick by the detection of a quantum jump^66,67^. Alternatively, ticks can be registered by capturing emitted photons with a microwave photodetector^68–71^, although this approach is currently constrained by photon-detection fidelity.

## Methods

Here, we provide additional detail regarding the continuum description of the wave-packet preparation, how to determine clock precision ${{{\mathcal{N}}}}_{\varSigma }$ and ${{{\mathcal{N}}}}_{\infty }$ from the Lindbladian, and how the precision-entropy scaling is derived. Furthermore, details are provided on how to obtain the master equation model of the main text from an extensible microscopic model, and finally, technical aspects regarding the robustness against perturbations are given.

### Hydrodynamical continuum limit

In the limit of large values for the number of ring sites *n*, the wave-packet propagation can be described using a continuum description, where we define the real space coordinates as *x*_*j*_ = *j*/*λ*_*ℓ*_ and go from the discrete coordinates *j* = 0, 1, …, *n* − 1 to the continuum $x\in \left[0,n/{\lambda }_{\ell }\right)\to {{\mathbb{R}}}_{+}$ in the limit *n* ≫ *λ*_*ℓ*_. The particle density $n(t,{x}_{j})={\lambda }_{\ell }^{-1}{| \left\langle j| \psi (t)\right\rangle | }^{2}$ can be described by the evolution equation

8 $${\partial }_{t}n(t,x)=-2{\partial }_{x}(g(x)n(t,x)),$$

where *g*(*x*) is defined by the coupling constant *g*(*x*_*j*_) = *g*_*j*_. The equation of motion for *n*(*t*, *x*) follows from the continuum limit of the Schrödinger equation $i{\partial }_{t}\left\vert \psi (t)\right\rangle =H\left\vert \psi (t)\right\rangle$, and conserves probabilities $1=\int_{0}^{\infty }{{\rm{d}}}x\,n(t,x)$. The continuum model is valid so long as we consider times *t* < *n*/(2*g*) where the wave packet has not yet reached the right ramp of the coupling potential (equation (6)), and furthermore *λ*_*ℓ*_, *n* ≫ 1, to ensure that *g*(*x*) and *n*(*t*, *x*) do not vary quickly on the lattice length scale. The derivation of the continuum model is discussed in further detail in Supplementary Section C1, where we also provide the analytical solution for *n*(*t*, *x*). The numerics confirm that the continuum limit captures well the scaling behaviour of the discrete wave function beyond the length scale of the lattice. In particular, we find that the initial distribution *n*(0, *x*) is transported to *x* > 0 and broadened by the width *λ*_*ℓ*_ of the initial ramp. This leads to the scaling form ${| \left\langle j| \psi (t)\right\rangle | }^{2} \sim {\lambda }_{\ell }^{-1}f{((j-2gt)/{\lambda }_{\ell })}^{2}$ of the wave packet in the limit of large times and and displacement *x*, 2*gt* ≫ *λ*_*ℓ*_, where *f* is some function independent of *n* and *λ*_*ℓ*_ describing the shape of the travelling wave packet.

### Clock precision with full counting statistics

The definitions brought forward in equations (4) and (5) of the main text defined clock precision relative to the tick number statistics ${{{\mathcal{N}}}}_{\varSigma }={\lim }_{t\to \infty }{{\rm{E}}}[N(t)]/{{\rm{Var}}}[N(t)]$ and relative to the waiting time statistics ${{{\mathcal{N}}}}_{\infty }={{\rm{E}}}{[T]}^{2}/{{\rm{Var}}}[T\,]$. Here, we provide the necessary details to calculate these two quantities based on the Lindblad master equation ${{\mathcal{L}}}={{{\mathcal{L}}}}_{0}+{{{\mathcal{L}}}}_{+}+{{{\mathcal{L}}}}_{-}$. The decomposition of the Lindbladian into three terms separates out the ‘no jump’ part

9 $${{{\mathcal{L}}}}_{0}\,\cdot \,=-i[H,\,\cdot \,]-\frac{1}{2}\{\,{J}^{{\dagger} }J,\,\cdot \,\}-\frac{1}{2}\{\,{\overline{J}}^{{\dagger} }\overline{J},\,\cdot \,\},$$

describing the non-Hermitian evolution conditioned on no jumps. The jumps generated by *J* defining positive ticks are described by ${{{\mathcal{L}}}}_{+}\,\cdot \,=J\cdot {J}^{{\dagger} }$, and the ones for $\overline{J}$, the negative ticks, by ${{{\mathcal{L}}}}_{-}\,\cdot \,=\overline{J}\cdot {\overline{J}}^{{\dagger} }$. The late time statistics of *N*(*t*) are fully determined by introducing the tilted Liouvillian^73^

10 $${{\mathcal{L}}}(\,\chi )={{{\mathcal{L}}}}_{0}+{{\mathrm{e}}}^{i\chi }{{{\mathcal{L}}}}_{+}+{{\mathrm{e}}}^{-i\chi }{{{\mathcal{L}}}}_{-},$$

with discrete counting field *χ*. The eigenvalue *λ*(*χ*) of ${{\mathcal{L}}}(\,\chi )$ that has the largest real value is equal to the cumulant generating function *t*^−1^*C*(*χ*, *t*) of *N*(*t*) at late times, and thus, we can determine the *k*th cumulant

11 $${\lim }_{t\to \infty }{t}^{-1}\langle \langle N{(t)}^{k}\rangle \rangle ={\left(-i{\partial }_{\chi }\right)}^{k}\lambda (\chi ){| }_{\chi = 0}.$$

The special cases *k* = 1, 2 give the expectation value and variance of *N*(*t*) needed to determine the clock precision ${{{\mathcal{N}}}}_{\varSigma },$ as further detailed in Supplementary Section D1.

In the limit where ${{{\mathcal{L}}}}_{-}=0$, that is, the limit of infinite entropy production, the counting variable *N*(*t*) is a renewal process^54^, that is, the time between each increment of *N*(*t*) is an independent and identical copy of the waiting time *T*. In this limit, the late time cumulants of *N*(*t*) are fully determined by the statistics of *T*, and one can relate^16,54^

12 $${\lim }_{t\to \infty }\frac{{{\rm{E}}}[N(t)]}{t}=\frac{1}{{{\rm{E}}}[T\,]},\quad {\lim }_{t\to \infty }\frac{{{\rm{Var}}}[N(t)]}{t}=\frac{{{\rm{Var}}}[T\,]}{{{\rm{E}}}{[T\,]}^{3}},$$

showing that $${{{\mathcal{N}}}}_{\varSigma }\to {{{\mathcal{N}}}}_{\infty }$$ as *Σ*_tick_ → *∞*. The waiting time statistics for $${{{\mathcal{L}}}}_{-}=0$$ can also be determined directly with the Liouvillian, $$E[{T}^{k}]$$$$={(-1)}^{k}k!{{\rm{tr}}}[{{{\mathcal{L}}}}_{0}^{\,-k}{\rho }_{0}]$$. A detailed derivation can be found in Supplementary Section B1.

As for the introductory statement that ${{{\mathcal{N}}}}_{\infty }$ equals how many times the clock ticks until it misses one tick compared with the perfect parameter time, it can be shown to be consistent with the definition ${{{\mathcal{N}}}}_{\infty }={{\rm{E}}}{[T\,]}^{2}/{{\rm{Var}}}[T\,]$ (ref. ^12^). As the time between ticks is independently and identically distributed, the variance of the time to the *k*th tick is given by Var[*T*_*k*_] = *k*Var[*T*]. Setting the standard deviation of the *k*th tick to equal the time between two ticks ${{\rm{Var}}}{[{T}_{k}]}^{1/2}\equiv {{\rm{E}}}[T\,]$, we can formally solve for *k* to give $k={{{\mathcal{N}}}}_{\infty }$, recovering the initial statement.

### Theory of the precision–entropy scaling

In case of finite entropy dissipation, the master equation is perturbed by a term describing the negative ticks, which is of magnitude $\delta ={{\mathrm{e}}}^{-{\varSigma }_{{{\rm{tick}}}}}$. In the limit of small *δ*, the clock precision can be expanded in powers of this correction, ${{{\mathcal{N}}}}_{\varSigma }={{{\mathcal{N}}}}_{\infty }+O(\delta )$, using the Landau big-*O* notation^35^. Because we aim to minimize entropy *Σ*_tick_, we want to keep *δ* as large as possible while at the same time making sure the precision ${{{\mathcal{N}}}}_{\varSigma }$ is sufficiently close to ${{{\mathcal{N}}}}_{\infty }$.

If *δ* = *n*^−*ζ*^ decays algebraically for some exponent *ζ* > 0 large enough to cancel out the constant factors in the big-*O* correction, the entropy scales only logarithmically while at the same time keeping the perturbations due to the negative ticks small. How large *ζ* needs to be is determined in part by the spectral gap of the system’s Lindbladian and by whether it scales algebraically with system size (which is related to the famously hard problem of undecidability of the existence of spectral gaps^74^; Supplementary Section D). We have numerically determined the scaling up to *n* = 200 and found that the choice *ζ* = 4 is sufficient to make the error between ${{{\mathcal{N}}}}_{\varSigma }$ and ${{{\mathcal{N}}}}_{\infty }$ arbitrarily small as *n* grows. Using the identity $\log \delta =-{\varSigma }_{{{\rm{tick}}}}$, we find the entropy scales logarithmically ${\varSigma }_{{{\rm{tick}}}}=\zeta \log n$, whereas ${{{\mathcal{N}}}}_{\varSigma } \sim {n}^{1.31}$ scales polynomially (with the negligible correction), which results in equation (1).

### Applicability of the master equation

Using spin chains or CCAs to model the ring clock from the main text leads to an effective description with the Hamiltonian *H* as in equation (2) and jump operators $J,\overline{J}$. Working with a spin chain, the local Hamiltonians are given by $\frac{\omega }{2}{\sigma }_{z,\,j}$ for site *j*, and the nearest-neighbour coupling can be modelled by the particle number conserving hopping term *g*_*j*_*σ*_−,*j*_*σ*_+,*j*+1_ + h.c. The challenging part for a microscopic description comes from the fact that we have to prevent multiple excitations entering the ring at once to ensure that the description we have used so far is valid. One way to solve this problem is to treat the first and the last site of the ring clock separately as a single system—the ticking element—as proposed in Fig. 1b. To ensure that we recover the effective dynamics described in the main text, this ticking element has to (1) remember when it has emitted an excitation into the ring such that it does not emit another excitation into the ring before it has ticked, and (2) it has to be boundary-matched to the ring couplings *g*_*j*_ to avoid the reflection of the incoming wave packet.

The proposed level scheme comprises five states and undergoes the following cycle for a tick: $\left\vert g\right\rangle \to \left\vert \ell \right\rangle \to \left\vert m\right\rangle \to \left\vert e\right\rangle \to \left\vert r\right\rangle \to \left\vert g\right\rangle$. Note, we identify $\left\vert \ell \right\rangle \equiv \left\vert n-1\right\rangle$ as formally the last ring site in terms of the states of the reduced model, and $\left\vert r\right\rangle \equiv \left\vert 0\right\rangle$ as the first site. The idea of this scheme is that, by adiabatically eliminating the intermediate states $\left\vert m\right\rangle$ and $\left\vert e\right\rangle$ from the tick cycle, we recover the jump process $\left\vert n-1\right\rangle \equiv \left\vert \ell \right\rangle \to \left\vert 0\right\rangle \equiv \left\vert r\right\rangle$ generated by *J* (and the inverse generated by $\overline{J}$). To get there, we look into each step of the tick cycle separately. When the excitation is in the ring, the ticking element is in the ground state $\left\vert g\right\rangle$. When the excitation arrives at the site $\left\vert n-2\right\rangle$ in the ring, it couples to the transition $\left\vert g\right\rangle \to \left\vert \ell \right\rangle$ with strength *g*_*n*−2_. In turn, the state $\left\vert \ell \right\rangle$ decays dissipatively as $\left\vert \ell \right\rangle \to \left\vert n\right\rangle$ with rate *Γ*, which defines the tick. This is the rate that crucially has to be boundary matched to the couplings *g*_*j*_, and the strength of the reverse process needs to be suppressed with the entropy production *Σ*_tick_ by coupling this transition to a cold enough bath at inverse temperature *β*_*C*_. After this decay, however, the system is not in resonance with the frequency *ω* of the ring structure anymore and it has to brought to a higher energy again, for which two additional dissipative drives can be used. With an additional hot bath at inverse temperature *β*_*H*_ driving $\left\vert m\right\rangle \leftrightarrow \left\vert e\right\rangle$ at a rate *κ*_*H*_ and an additional cold bath at inverse temperature *β*_*C*_ mediating $\left\vert e\right\rangle \to \left\vert r\right\rangle$ at a rate *κ*_*C*_, we can create a population inversion to ensure that, in the end, the state that arrived on the ring ends up on $\left\vert r\right\rangle$. Then, the ticking element is again on resonance with *ω*, and the excitation can be coherently coupled to the next site with strength *g*_0_, completing the cycle.

When the excitation is in the ring, the ticking element latches to the ground state $\left\vert g\right\rangle$, which is not addressed by the thermal drives, and therefore, no second excitation is emitted into the ring. So long as *ω* ≫ *κ*_*H*_, *κ*_*C*_, *Γ* ≳ *g*_*j*_ is the hierarchy of energy scales in the problem, the Lindblad master equation is applicable in a local picture with weak interactions despite the many-body nature^75,76^, and the notion of entropy *Σ*_tick_ we used coincides with the thermodynamic entropy from Clausius’ law^77^ as in equation (3).

### Stability under perturbations

In a realistic experimental implementation of the ring clock, the actual nearest-neighbour couplings would differ slightly from the ideal choice *g*_*j*_. From the disorder in the couplings, we would expect the clock to perform worse due to imperfect preparation of the travelling wave packet in the preparation region, off-diagonal Anderson localization in the bulk^78–81^ and reflection in the final region of the ring. Furthermore, in a realistic scenario, the bulk of the ring would not be perfectly isolated from the environment. As a result, the bulk sites may absorb excitations from the environment, or lose them, negatively impacting the precision. We analyse the stability of the main result equation (1) under these two perturbations of the ideal scenario. In a concrete experimental realization, further sources of error may contribute depending on the details of the physical platform, such as disorder in the bare qubit frequencies in superconducting circuits, whose impact we expect to be comparable to that of imperfections in the couplings *g*_*j*_.

For a fixed but small error *ε*_loc_ in the nearest-neighbour couplings or an unwanted coupling *ε*_env_ to the environment, the predicted precision scaling from equation (7) can be upheld up to some finite ring length ${n}_{\max }$. For longer rings, the errors start to dominate and the precision decreases again (Extended Data Figs. 2a and 3a). The analysis in the following shows that this point of breakdown ${n}_{\max }$ of the optimal scaling increases the smaller the error *ε* in the couplings and dissipation is with the precise relationship of the form

13 $${n}_{\max } \sim \frac{1}{{\varepsilon }^{\kappa }},$$

where *κ* > 0 is some exponent that depends on the type of error. This relationship (equation (13)) is algebraic rather than exponential, and in the following we determine the exponents *κ*_loc_ for the parameter perturbations as well as *κ*_env_ for the environmental perturbation seperately.

### Disorder in the couplings

The error model for the couplings *g*_*j*_ perturbs each nearest-neighbour coupling with a multiplicative factor ${(1+{\varepsilon }_{{{\rm{loc}}}})}^{\hat{{X}}_{j}}$, where *ε* > 0 is the error and ${\hat{X}}_{j} \sim {{\mathcal{N}}}(0,1)$ is normally distributed with mean 0 and variance 1. To create Extended Data Fig. 2a, for a fixed ring length *n* and error *ε*, *M* = 400 independently and identically distributed (i.i.d.) samples of perturbed couplings ${\tilde{g}}_{j}$ are generated, leading to a perturbed precision value ${\tilde{{{\mathcal{N}}}}}_{\infty }$. Then, a statistical average is taken ${\langle {\tilde{{{\mathcal{N}}}}}_{\infty }\rangle }_{\hat{X}}$ over the *M* random choices of disordered parameters ${\tilde{g}}_{j}$. It can be seen that, for *ε*_loc_ fixed, this average value increases until a saddle point ${n}_{\max }$ is reached and the precision decreases again. If we fit a linear slope (black dashed line) in the log–log scale through the maxima of the average curves for all error values, we find that the precision still scales as ~*n*^1.39(5)^, albeit with a constant factor smaller in comparison with the optimal scaling. This scaling approximately agrees with the ideal one. The uncertainty in the exponent comes from the distribution of the numerically determined maxima and is determined with the covariance matrix from the linear regression. Extended Data Fig. 2b shows how this maximal length ${n}_{\max }$ changes as a function of the *ε*_loc_ and a linear fit in the log–log plot reveals a scaling exponent *κ*_loc_ = 0.81(3) for equation (13). The uncertainty in the exponent comes from the statistical distribution of the data points ${n}_{\max }$ and is determined again using the covariance matrix from the linear fit. Note that the maximum ring length in this analysis is of the order *n* = 300 owing to limitations in the computational runtime for the statistical averaging.

### Undesired environment interactions

In the ideal clock model, the ticks are generated by $J=\sqrt{\varGamma }\left\vert 0\right\rangle \left\langle n-1\right\vert$. To model erroneous losses due to the environment along the ring in the spirit of the abstract model, we assume that these decays are also registered as ticks leading to a premature reinitialization of the clock akin to the tick process defined by the jump operator *J*. This lowers the expected time between two ticks E[*T*] and increases the variance Var[*T*], thus overall decreasing the clock precision ${{{\mathcal{N}}}}_{\infty }$. For an actual experimental realization of the ring clock, this description is of course only an approximation of how losses would impact the result, but it captures the main features of such imperfections. We include these processes with additional dissipators in the master equation description ${L}_{j}=\sqrt{\varGamma {\varepsilon }_{{{\rm{env}}}}}\left\vert 0\right\rangle \left\langle j\right\vert$ for sites *j* = 1, …, *n* − 2, where *ε*_env_ > 0 is the fractional error in the dissipation. For a finite-temperature environment, however, also the reverse process ${L}_{j}^{{\dagger} }$ is possible. Due to detailed balance, the rate of this process is suppressed by a factor ${{\mathrm{e}}}^{-{\varSigma }_{{{\rm{env}}}}}$, where *Σ*_env_ is the entropy production in the (finite temperature) environment. More importantly, it may also happen that the bulk absorbs a second excitation from the environment, leading to two excitations in the spin chain. If the ring is initially occupied only on site *j* = 0, …, *n* − 1, an absorption on any other site *i* ≠ *j* can be modelled by the jump operator

14 $${L}_{(ji),\,j}={{\mathrm{e}}}^{-{\varSigma }_{{{\rm{env}}}}/2}\sqrt{\varGamma {\varepsilon }_{{{\rm{env}}}}}\left\vert\,j,i\right\rangle \left\langle j\right\vert ,$$

where $\left\vert\,j,i\right\rangle$ is the state with exactly two excitations, one on each site *i* and *j*. Detailed balance guarantees the existence of the reverse process in which the doubly occupied state $\left\vert\,j,i\right\rangle$ loses an excitation into the environment. This excitation can be lost on both sites *i* and *j*. For example, if site *i* decays, the jump operator is given by ${L}_{j,(ji)}=\sqrt{\varGamma {\varepsilon }_{{{\rm{env}}}}}\left\vert\,j\right\rangle \left\langle j,i\right\vert$. Although, in principle, higher excitation numbers >2 in the ring would be possible, a sufficiently cold environment exponentially suppresses the thermal occupation probability of these higher subspaces. In this context, already a logarithmically growing inverse temperature ${\beta }_{{{\rm{env}}}} \sim \log n$ of the environment is sufficient. At the same time, an environment temperature with such scaling ensures that the entropy produced by an excitation ${\varSigma }_{{{\rm{env}}}}={\beta }_{{{\rm{env}}}}\omega \sim \log n$ grows logarithmically only in the ring length (for fixed bare spin frequency *ω*). An exact and effective treatment of the single- and double-excitation subspaces thus captures the dominant error contributions to the clock precision (see details in Supplementary Section E).

In Extended Data Fig. 3a, we show how the precision ${{{\mathcal{N}}}}_{\varSigma }$ scales as a function of the ring length, *n*, for a selection of fixed errors *ε*_env_, and with entropy ${\varSigma }_{{{\rm{env}}}}=\frac{3}{2}{\varSigma }_{{{\rm{tick}}}}$ also only logarithmically growing with chain length. For up to *n* = 12 sites, we numerically simulate the full dynamics in the double-excitation subspace, while for 12 ≤ *n* ≤ 200 we use a computationally efficient, effective model for extrapolation (see details in Supplementary Section E). The inset in Extended Data Fig. 3a shows that the difference between exact and effective model vanishes for growing *n*, supporting the applicability of the effective model. Similar to the coupling perturbations we find that, for every fixed error *ε*_env_, there is a turn-around point ${n}_{\max }$, where the precision reaches its maximum and after which the precision decreases as the ring gets longer. A fit (dashed black line) through all the maxima recovers a polynomial scaling of the precision, ~*n*^1.23(1)^. Together with ${\varSigma }_{{{\rm{env}}}} \sim \log n$, this still guarantees an exponential precision–entropy relationship, albeit with a lower exponent than in the idealized case. We show the log–log relationship between *ε*_env_ and ${n}_{\max }$ in Extended Data Fig. 3b, where a linear fit reveals a an exponent *κ*_env_ = 0.43 following equation (13). We further observe that the ring clock is more sensitive to amplitude damping noise in the bulk than to disorder in the couplings, as the tolerable loss *ε*_env_ is smaller than the coupling error *ε*_loc_; yet, in both cases, the scaling with ${n}_{\max }$ remains algebraic.

## Online content

Any methods, additional references, Nature Portfolio reporting summaries, source data, extended data, supplementary information, acknowledgements, peer review information; details of author contributions and competing interests; and statements of data and code availability are available at 10.1038/s41567-025-02929-2.

## Supplementary information

Supplementary Information Supplementary technical details and Supplementary Figs. 1–6.

## Data Availability

All parameters and data are available via GitHub at https://github.com/aspects-quantum/ring-clock.

## Code availability

All code is available via GitHub at https://github.com/aspects-quantum/ring-clock.
