# Supplementary material for: Precision is not limited by the second law of thermodynamics
Source: Nat Phys. 2025 Jun 2;21(7):1147–52. doi: 10.1038/s41567-025-02929-2 (PMC12263443; doi:10.1038/s41567-025-02929-2)
Supplement: Supplementary file 1 — Supplementary technical details and Supplementary Figs. 1–6. [file 41567_2025_2929_MOESM1_ESM.pdf]

nature physics

Supplementary information

https://doi.org/10.1038/s41567-025-02929-2

# Precision is not limited by the second law of thermodynamics

In the format provided by the authors and unedited

## CONTENTS


A. Calculating the $g^{(2)}$-function 1

B. Details on the numerical optimization of the precision 1
  1. Clock precision 1
  2. Maximizing clock precision for infinite entropy production 2

C. Theory of clock precision in the limit of infinite entropy production 3
  1. Preparation 3
  2. Bulk propagation 5
  3. Emission 6
    a. Apodization of the right ramp 6
    b. Scaling of clock precision with ring length 9

D. Exponential scaling of precision with entropy production 11
  1. Calculating clock precision in the reversible regime 11
  2. Clock precision at finite entropy production 12

E. Amplitude damping noise in the bulk 14

References 16


## Appendix A: Calculating the $g^{(2)}$-function

Here we provide pertinent detail for how the autocorrelation function $g^{(2)}(\tau)$ shown in Fig. 2 of the main text is calculated. For a current operator $I = J^\dagger J$ and in the steady-state limit, it is defined as

$$g^{(2)}(\tau) = \lim_{t\to\infty} \frac{\mathrm{E}[I(t+\tau)I(t)]}{\mathrm{E}[I(t)]^2}. \tag{A1}$$

We work in the regime with infinite entropy production, that is, no backwards ticks $\overline{J} = 0$ and thus, the Liouvillian is given by $\mathcal{L}\cdot = -i[H,\cdot] + J\cdot J^\dagger - \frac{1}{2}\left\{J^\dagger J,\cdot\right\}$. The autocorrelation function can then be explicitly determined using the expression (see e.g. Ref. [1]),

$$\mathrm{E}[I(t+\tau)I(t)] = \mathrm{tr}\left[J^\dagger J e^{\mathcal{L}\tau}\left(J\rho(t)J^\dagger\right)\right], \tag{A2}$$

where $\rho(t) \to \rho_{\mathrm{s.s.}}$ in our case is taken to be in the steady-state limit. Similarly, we can determine

$$\mathrm{E}[I(t)] = \mathrm{tr}\left[J^\dagger J\rho(t)\right], \tag{A3}$$

which together allows us to calculate the $g^{(2)}$-function as in eq. (A1). Since by construction of the idealized model, the ring clock works in the single-excitation subspace, we have $g^{(2)}(0) = 0$.

## Appendix B: Details on the numerical optimization of the precision

In the following, we provide a more in-depth derivation of how it is possible to calculate $\mathcal{N}_\infty$ given the dynamical description of the clock, that is the Hamiltonian $H$ and the tick generating operator $J$ (Sec. B 1), and how the precision is maximized (Sec. B 2). As for how $\mathcal{N}_\Sigma$ and $\mathcal{N}_\infty$ are related, we will answer this question in detail in Sec. D.

### 1. Clock precision

Here, we work in the fully irreversible regime without the reverse jump process $\overline{J}$, and as pointed out in the Methods, clock precision in this limit can be calculated with the waiting time statistics

$$\mathcal{N}_\infty = \frac{\mathrm{E}[T]^2}{\mathrm{Var}[T]^2}. \tag{B1}$$

This quantity has thus been the standard measure for clock precision in the literature [2–5], and can be interpreted as the number of times the clock ticks on average until it goes off by one compared to a perfect reference clock [2].

We now show how to calculate $\mathrm{E}[T]$ and $\mathrm{Var}[T]$. Without reverse ticks, the equations of motion are defined by the Liouvillian $\mathcal{L} = \mathcal{L}_0 + \mathcal{L}_+$, where the term $\mathcal{L}_0\cdot = -i[H,\cdot] - \frac{1}{2}\left\{J^\dagger J,\cdot\right\}$ is the conditional non-trace-preserving part of the evolution where no tick occurs, and $\mathcal{L}_+\cdot = J\cdot J^\dagger$ is the part generating the ticks. Given some initial state of the clock, $\rho_0$, the non-normalized evolution given that the clock has not yet ticked is generated by $\mathcal{L}_0$, and, by taking the trace, we can find the survival probability $P[T\geq t]$ that at time $t$ the tick has not yet occurred [6, 7],

$$P[T\geq t] = \mathrm{tr}\left[e^{\mathcal{L}_0 t}\rho_0\right]. \tag{B2}$$

Under the assumption that the resulting probability density function (PDF) $p(t) = P[T=t]$ is normalized, that is $\lim_{t\to\infty} P[T\geq t] = 0$, we can calculate all moments of $T$ and thereby also the desired quantities $\mathrm{E}[T]$ as well as $\mathrm{Var}[T]$ needed determine the clock precision $\mathcal{N}_\infty$. Upon integration over $t\in\mathbb{R}_+$, we get

$$\mathrm{E}[T^k] = k\int_0^\infty \mathrm{d}t\, t^{k-1}\,\mathrm{tr}\left[e^{\mathcal{L}_0 t}\rho_0\right] \tag{B3}$$

$$= (-1)^k k!\,\mathrm{tr}\left[(\mathcal{L}_0)^{-k}\rho_0\right], \tag{B4}$$

where the expression $\rho_k = (\mathcal{L}_0)^{-k}\rho_0$ can be obtained by recursively solving a Lyapunov equation [4]. To be explicit, the equation $\rho_k = (\mathcal{L}_0)^{-1}\rho_{k-1}$ is equivalent to

the continuous-time Lyapunov equation

$$\underbrace{\left(-iH+\frac{1}{2}J^\dagger J\right)}_{=:-iH_{\text{eff}}}\rho_k+\rho_k\left(-iH+\frac{1}{2}J^\dagger J\right)^\dagger=\rho_{k-1}. \tag{B5}$$

This type of equation arises for when considering stability in quantum master equations [8, 9] and classical linear control theory [10]. Here, we can also identify the non-Hermitian effective Hamiltonian $H_{\text{eff}}$ as already in the main text. To calculate the precision fraction $\mathrm{E}[T]^2/\mathrm{Var}[T]$, only the first two iterations are required, and in shortened notation, we can write

$$\mathcal{N}_\infty=\left(\frac{2\,\mathrm{tr}\left[(\mathcal{L}_0)^{-2}\rho_0\right]}{\mathrm{tr}\left[(\mathcal{L}_0)^{-1}\rho_0\right]^2}-1\right)^{-1}. \tag{B6}$$

For our considerations, we use the initial state $\rho_0=|0\rangle\langle 0|$, which is the same state the ring clock resets to after a tick $\mathcal{L}_+(\rho)\propto|0\rangle\langle 0|$ regardless of the state $\rho$ before the tick.

When considering the survival probability $P[T\geq t]$, it is advantageous to use the purity of the initial (reset) state so the evolution decomposes $e^{\mathcal{L}_0 t}|0\rangle\langle 0|=e^{-iH_{\text{eff}}t}|0\rangle\langle 0|\,e^{iH_{\text{eff}}^\dagger t}$ which requires the exponentiation of a matrix only of size $O(n^2)$ instead of $O(n^4)$. Then, the tick probability density is given by

$$P[T=t]=-\frac{\mathrm{d}}{\mathrm{d}t}\underbrace{\mathrm{tr}\left[e^{\mathcal{L}_0 t}|0\rangle\langle 0|\right]}_{=P[T\geq t]} \tag{B7}$$

$$=\Gamma\left|\langle n-1\left|e^{-iH_{\text{eff}}T}\right|0\rangle\right|^2. \tag{B8}$$

### 2. Maximizing clock precision for infinite entropy production

The clock precision $\mathcal{N}_\infty$ for fixed initial state $\rho_0=|0\rangle\langle 0|$ is a function of the parameters $g_j$ in the Hamiltonian $H=H[\{g_j\}]$ and the ticking rate $\Gamma$ in the jump operator $J$. Precision is dimensionless $[\mathcal{N}_\infty]=1$, and thus $\mathcal{N}_\infty$ is invariant under an arbitrary re-scaling of all the rates. To simplify the analysis, we may therefore fix the parameter $\Gamma$ to some value for the optimization and the remaining parameters $\{g_j\}$ are varied in units of $\Gamma$. For the most general case we would of course like to determine the global optimum, maximizing over all possible couplings $\{g_j\}_{j=0}^{n-2}$ on the ring,

$$\{g_j\}=\underset{g_j}{\mathrm{argmax}}\{\mathcal{N}_\infty\}. \tag{B9}$$

This global optimization of the couplings, however becomes prohibitive $O(n)$ for large system sizes, while for small values of $n\lesssim 50$ the numerical optimization is stable and converges to the global minimum. From this solution we extract the ansatz for $g_j$ displayed in Eq. (6) of the main text or

$$g_j=-\mu_\ell e^{-j/\lambda_\ell}+g+\mu_r e^{(j-(n-1))/\lambda_r}, \tag{B10}$$

which we repeat here for the reader's convenience. For system sizes beyond $n>50$, we used the ansatz in eq. (B10). In this case, it is not guaranteed that we find the global maximum of $\mathcal{N}_\infty$, but since the ansatz in eq. (B10) does not include the ring size $n$ any more, the optimization of

$$(\mu_\ell,\mu_r,g,\lambda_\ell,\lambda_r)=\underset{\{\mu_\ell,\mu_r,g,\lambda_\ell,\lambda_r\}}{\mathrm{argmax}}\{\mathcal{N}_\infty\}, \tag{B11}$$

is greatly simplified because only 5 parameters need to be optimized. Our numerical simulation shows for values $n\lesssim 50$ where both optimization algorithms converge, that the two methods yield qualitatively similar shapes for the couplings $g_j$, where there are the three distinct regions: (1) initial ramp, (2) flat bulk couplings and (3) emission ramp, as we show in Fig. 1(a). Furthermore, also the maximal clock precision deviates only negligably between the two cases as visualized in Fig. 1(b). This may not be too surprising since the optimization is initialized for small system sizes $n$ where we trust eq. (B10) to be the global optimum and then iteratively the ring length is increased $n\to n+1$ as well as the optimum of the previous $n$ is seeded as initial state for the optimization for $n+1$. Since we expect the optimization landscape to change almost continuously from $n\to n+1$, we expect that our optimization remains close to the global optimum.

The optimal parameters for $\mu_\ell,\mu_r,g,\lambda_\ell$ and $\lambda_r$ are shown in the Extended Data Fig. 1. We find that $\lambda_\ell\sim n^{0.35}$ scales approximately as our predicted power law $n^{1/3}$ from the main text. Furthermore we find that $\lambda_r$ as well as $\mu_r$ do not scale with the ring size for $n\gg 1$.

One way to numerically solve the Lyapunov equation of this problem is to start by diagonalizing the effective Hamiltonian. By writing $V^{-1}(-iH_{\text{eff}})V=\Lambda$, where $\Lambda$ is a diagonal matrix, we can rewrite the Lyapunov recursion relation from eq. (B5) in the following way,

$$V^{-1}\rho_k V^\dagger=\Lambda V^{-1}\rho_{k+1}V^\dagger+V^{-1}\rho_{k+1}V^\dagger\Lambda^\dagger. \tag{B12}$$

By defining $\sigma_k=V^{-1}\rho_k V^\dagger$, the Lyapunov equation further reduces to

$$\sigma_k=\Lambda\sigma_{k+1}+\sigma_{k+1}\Lambda^\dagger, \tag{B13}$$

which is solved in $O(n^2)$ steps because $\Lambda$ is diagonal. It is possible to rewrite this equation element by element, $(\sigma_k)_{mn}=(\lambda_m+\lambda_n^*)(\sigma_{k+1})_{mn}$, which directly yields $\sigma_{k+1}$. Given the diagonalization of $-iH_{\text{eff}}$ with the right and left eigenvectors encoded in $V$ and $V^{-1}$ respectively, we can then recover $\rho_k=V\sigma_k {V^{-1}}^\dagger$. In case we are interested in obtaining several moments of the waiting time distribution, we need to solve multiple iterations of the Lyapunov equation. This method has a better

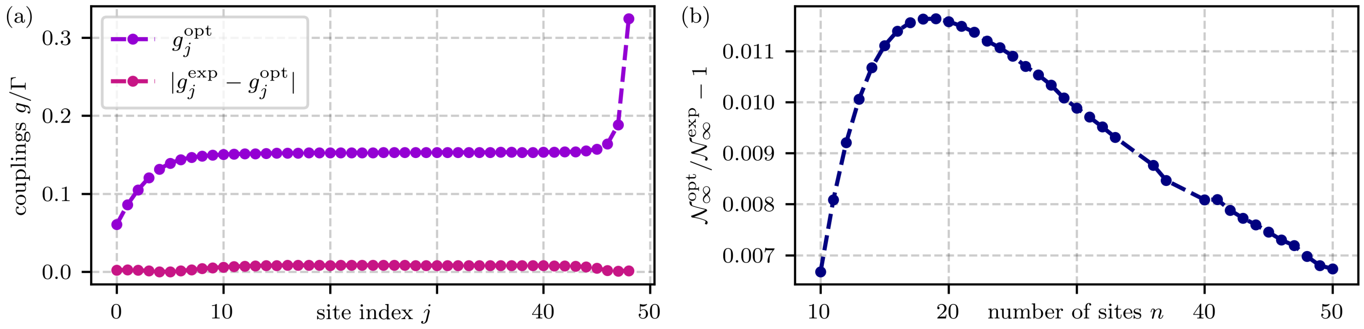


FIG. 1. In this panel we show the comparison between the true global optimum of coupling parameters $g_j$ and the optimum for the case where the coupling parameters are given by (B10). In (a), we show the difference between the two coupling parameters for the examplary case of $n = 50$ ring sites. The difference between the optimal couplings and the ones obtained using the exponential model is at most of order $10^{-2}$ in the bulk and on the boundaries the difference becomes even smaller, i.e., of the order of $10^{-3}$. In (b), we plot how the clock precision $\mathcal{N}_\infty$ differs for those two models. We see that the relative difference vanishes for large site numbers. Several outliers due to the numerical optimization have been removed for the site numbers between $n = 30$ and $n = 40$. A comparison becomes unfeasible for higher site numbers due to computational constraints in finding the global optimum with more than 50 parameters. In comparison, the exponential model requires the optimization of only 5 parameters regardless of the ring length.

scaling because the standard Bartels-Steward Lyapunov solution method using a QR decomposition uses $O(n^3)$ steps [11]. Particularly if $-iH_{\rm eff}$ can be efficiently diagonalized, using the recursive relation in (B13) can be more efficient in terms of the computational runtime required compared to using a standard Bartels-Steward Lyapunov solver [11].

## Appendix C: Theory of clock precision in the limit of infinite entropy production

In this section we assemble all the arguments to understand one of the main results of this works: the scaling of precision at infinite entropy production with system size,

$$\mathcal{N}_\infty \sim n^{4/3} \tag{C1}$$

as given in eq. (7) of the main text. The waiting time distribution leading to this result sensitively depends on the propagation of a single localized excitation through the spin chain with couplings given in eq. (6) of the main text. Accordingly, the propagation can be divided into three parts; (1) preparation (see Sec. C 1), (2) bulk propagation (see Sec. C 2) and (3) emission (see Sec. C 3), which are discussed separately in the main text. Combining all this leads to the desired scaling relation in eq. (C1) and eq. (7) respectively, to which we supply the details in Sec. C 3 b.

### 1. Preparation

The starting point of the system is the state $|0\rangle$, because after every tick, the system is reset to that state $|0\rangle$. Without loss of generality we can chose the initial time as $t = 0$. This state then evolves according to $|\psi(t)\rangle = e^{-iH_0 t}|0\rangle$ with the Hamiltonian

$$H_0 = \sum_{j=0}^{n-2} g_j\, |j\rangle\langle j+1| + \text{h.c.} \tag{C2}$$

Since we are only interested in the propagation through the initial ramp we set $g_j = -\mu_\ell e^{-j/\lambda_\ell} + g$, neglecting the effects of the second ramp from eq. (6) and from the dissipator $J$, because $J^\dagger J \propto |n-1\rangle\langle n-1|$ acts only on the last site. Contributions from $\overline{J}$ also play no role because we work in the limit of infinite entropy production for now. Propagating the excitation through this exponential ramp will then prepare the wave packet subsequently travelling through the bulk of the ring which is discussed below in Sec. C 2.

Since the Hamiltonian in eq. (C2) possesses neither translational invariance nor any other structure (e.g. of Töplitz form) allowing for a closed solution we will instead tread it approximately in the continuum with the hydrodynamic limit. To that end, large rings and soft ramps have to be considered $n, \lambda_\ell \gg 1$, where the wave functions barely varies on the scales of a single lattice spacing. This allows us to find an approximate continuum description of the Schrödinger equation on the lattice. In this limit all effects which are attributed to the lattice are being neglected. Writing the Schrödinger equation $i\partial_t|\psi(t)\rangle = H_0|\psi(t)\rangle$ element by element we obtain

$$i\partial_t\psi_j(t) = g_j\psi_{j+1}(t) + g_{j-1}\psi_{j-1}(t), \tag{C3}$$

where $\psi_j(t) = \langle j|\psi(t)\rangle$ is the amplitude of the wave function at site $j$. This equation is simplified by the canonical transformation $|\tilde{\psi}(t)\rangle = U|\psi(t)\rangle$, where

$$U = \sum_{j=0}^{n-1} e^{i\frac{j\pi}{2}}\, |j\rangle\langle j|\,. \tag{C4}$$

Elementwise, the transformation reads $\tilde{\psi}_j(t) = i^j \psi_j(t)$ which is reminiscent of targeting the hydrodynamic mode in relativistic Dirac fermions in one dimension which yields the Luttinger liquid [12, 13]. This canonical transformation renders the Schrödinger equation (C3) completely real

$$\partial_t \tilde{\psi}_j(t) = -g_j \tilde{\psi}_{j+1}(t) + g_{j-1} \tilde{\psi}_{j-1}(t). \tag{C5}$$

Note that the fully localized initial condition $\psi_j(0) = \delta_{j,0}$ is left untouched by this transformation $\tilde{\psi}_j(0) = \delta_{j,0}$. Since the initial condition is real so is $\tilde{\psi}_j(t)$ for all times $t \geq 0$. For simplicity, we will set $\tilde{\psi}_j \to \psi_j$ in the following. In order to perform the continuum limit we consider an equidistant lattice at $x_j = j/\lambda_\ell$ on the real line, where $j = 0, \ldots, n-1$.

The continuum limit of the discrete lattice wave function is done by finding continuous functions $\Psi(t, x)$ and $g(x)$ such that

$$\psi_j(t) \equiv \frac{1}{\sqrt{\lambda_\ell}} \Psi(t, x_j), \text{ and } g_j \equiv g(x_j) \tag{C6}$$

coincide. In the limit $\lambda_\ell, n \to \infty$, this can be achieved by identifying the continuum generalization $g(x) = -\mu_\ell e^{-x} + g$ for the couplings, and hydrodynamic limit of the Schrödinger equation in eq. (C5) giving

$$\partial_t \Psi(t, x) = -\frac{1}{\lambda_\ell} \left(2g(x)\partial_x \Psi(t, x) + (\partial_x g(x))\Psi(t, x)\right). \tag{C7}$$

To check the consistency of this expression with the discrete equation we first identify the finite differences in the discrete case with partial derivatives in the continuum case,

$$\partial_x g(x_j) = \frac{g_{j+1} - g_j}{\lambda_\ell} + O\left(\lambda_\ell^{-2}\right), \tag{C8}$$

and,

$$\partial_x \Psi(x_j, t) = \frac{\psi_{j+1}(t) - \psi_j(t)}{\lambda_\ell} + O(\lambda_\ell^{-2}). \tag{C9}$$

Inserting the identifications of eqs. (C8) and (C9) into eq. (C7) recovers eq. (C5) up to terms of order $O(\lambda_\ell^{-2})$. As for the normalization in the continuum $1 = \int_0^\infty dx |\Psi(x, t)|^2$, going to a Riemann sum shows it is compatible with the normalization of the discrete wave function $\sum_{j=0}^{n-1} |\psi_j(t)|^2 = \sum_{j=0}^{n-1} \frac{1}{\lambda_\ell} |\Psi(t, x)|^2 \to 1$, in the limit of $n, \lambda_\ell \gg 1$.

So long as both $\Psi(t, x)$ and $g(x)$ vary slowly on the length scale of the lattice, this approximation is good and the error can be controlled with the limit $\lambda_\ell \gg 1$. We can thus approximate the evolution of $|\psi(t)\rangle = \sum_j \psi_j(t)|j\rangle$ using the continuum limit $\Psi(t, x)$. In order to assess whether $\Psi(t, x)$ may be really interpreted as a wave function even for finite $\lambda_\ell$ and $n$ we have to ensure that $n(t, x) = |\Psi(t, x)|^2$ is a probability density and that $\int_0^\infty \mathrm{d}x\, n(t, x) = 1$ remains normalized exactly for all times. Using eq. (C7) we derive an effective equation of motion for $n(t, x)$ which gives

$$\partial_t n(t, x) = -\frac{1}{\lambda_\ell} \partial_x \left(2g(x) n(t, x)\right). \tag{C10}$$

This equation has the form of a transport equation [14] well studied in hydrodynamics. Since the left hand side is a total derivative with respect to $x$, integrating both sides shows that the integral of $n(t, x)$ will not change and that $\Psi(t, x)$ can indeed be treated as a wave function and eq. (C7) is a valid Schrödinger equation.

Both the equations for $\Psi(t, x)$ in (C7) and for $n(t, x)$ in (C10) can be solved analytically using the method of characteristics [14], a standard tool for studying first order partial differential equations (PDE). Here, we focus on the evolution of the probability density $n(t, x)$, which is the relevant quantity describing the transport of the wave packet and subsequently the waiting time distribution. For the analytical solution it is advantageous to expand the derivative equation (C10) which gives

$$\partial_t n(t, x) + \frac{2}{\lambda_\ell} g(x) \partial_x n(t, x) = -\frac{2}{\lambda_\ell} (\partial_x g(x)) n(t, x). \tag{C11}$$

This is solved analytically by the characteristic

$$\xi(t, x) = \log\left(\frac{\mu_\ell}{g}\left(1 - e^{-\frac{2gt}{\lambda_\ell}}\right) + e^{x - \frac{2gt}{\lambda_\ell}}\right), \tag{C12}$$

and expressing eq. (C11) as a function of $(t, \xi)$ instead of $(t, x)$. We then relate the total $t$-derivative of the density $n$ expressed in $(t, \xi)$ coordinates to eq. (C11) by making the identification $\partial_t x(t, \xi) = \frac{2}{\lambda_\ell} g(x(t, \xi))$. The PDE for $n(t, x)$ then turns into an ordinary differential equation that can be solved exactly. For an initial condition $n(0, x) = p(x)$ whose support is constrained to the positive reals $\mathbb{R}_+$, we get the following exact solution

$$n(t, x) = \frac{p(\xi(t, x))}{1 + \frac{\mu_\ell}{g}\left(e^{\frac{2gt}{\lambda_\ell} - x} - e^{-x}\right)}. \tag{C13}$$

With this solution we can study the asymptotic state $n(t, x)$ long after it has left the inital ramp $2gt \gg \lambda_\ell$. In this limit, we obtain

$$\xi(t, x) = \log\frac{\mu_\ell}{g} + \log\left(1 + \frac{g}{\mu_\ell} e^{x - \frac{2gt}{\lambda_\ell}}\right) + O\left(e^{-\frac{2gt}{\lambda_\ell}}\right). \tag{C14}$$

and the denominator in (C13) becomes a function of $x - 2gt/\lambda_\ell$.

We continue by considering the real-space asymptotics of the solution in eq. (C13) in the regime $x \gg 2gt/\lambda_\ell$ and $x \ll 2gt/\lambda_\ell$, still for times where the wave packet has left the initial ramp. We find that for large values of $x$, the initial distribution $p(x)$ of the wave packet dominates, because $\xi(t, x) = x - 2gt/\lambda_\ell + O(e^{-(x - 2gt/\lambda_\ell)})$,

and the denominator goes to 1 in that limit. For small values of $x$, on the other hand, we find $\xi(t,x) = \log \frac{\mu_\ell}{g} + O(e^{-(2gt/\lambda_\ell - x)})$ goes to a constant and the exponential from the denominator dominates. Both together yield the asymptotics of

$$n(t,x) = \begin{cases} p\left(x - \frac{2gt}{\lambda_\ell}\right), & x \gg \frac{2gt}{\lambda_\ell}, \\ \frac{g}{\mu_\ell} p \left(\log \frac{\mu_\ell}{g}\right) e^{-\left(\frac{2gt}{\lambda_\ell} - x\right)}, & x \ll \frac{2gt}{\lambda_\ell}. \end{cases} \tag{C15}$$

Therefore, asymptotically, the wave packet decays exponentially on its back side pointing away from the direction of propagation. The asymptotic behavior on its front side towards the direction of propagation is given by the shape of the initial state $p(x)$.

Finally we study the eq. (C13) in the limit where $2gt$ is comparable with $\lambda_\ell$ as well as $x \gg 1$. In this limit the solution can be cast in the form

$$n(t,x) = f\left(x - \frac{2gt}{\lambda_\ell}\right)^2 + O\left(e^{-\frac{2gt}{\lambda_\ell}} \partial_x p\right), \tag{C16}$$

with some function $f$ dependent on the initial state density $p$ which corresponds to the wave function $\Psi(t,x) = f(x - 2gt/\lambda_\ell)$. The initial state on the lattice is perfectly localized in $|0\rangle$ in real space. An infinitely localized state as an initial condition is of course at odds with our effective hydrodynamic description but we will initialize a wave packet in a $p(x)$ which is strongly concentrated on hydrodynamics scales (for example a Gaussian). While this treatment is very crude, the numerics in Fig. 4(a) suggest that the relevant quantity below, the width, is well captured in this approach, even though the hydrodynamic approximation looses quickly oscillating effects on the lattice length scale. The main result of this section is the preparation of the wave packet in the form (C16), which will turn out to be crucial to obtain the scaling of the clock precision.

### 2. Bulk propagation

In Sec. C 1, we studied the asymptotic form of the wave packet long after it has left the initial ramp using a hydrodynamics description. We will now analyze how the wave packet propagates through bulk of the ring. This limit we describe in terms of the effective translationally invariant Hamiltonian

$$H_{\text{bulk}} = \sum_{j=0}^{n-1} g\, |j+1\rangle\langle j| + \text{h.c.}, \tag{C17}$$

in good agreement with $g_j$ from eq. (6) in the limit where $n - \lambda_r \gg j \gg \lambda_\ell$. Leveraging the translational invariance of eq. (C17), we go from a basis of localized states on the lattice $|j\rangle$ to momentum space $|\psi_{k_\ell}\rangle$. This is given by

$$|\psi_{k_\ell}\rangle := \frac{1}{\sqrt{n}} \sum_{j=0}^{n-1} e^{-ik_\ell j} |j\rangle, \tag{C18}$$

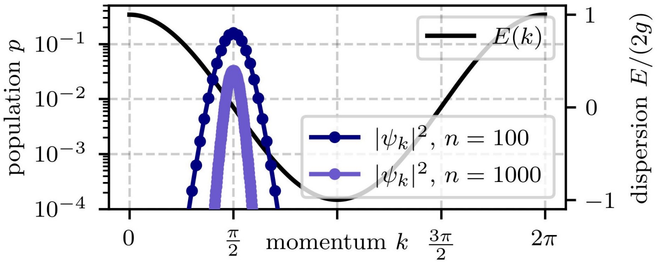


FIG. 2. Dispersion relation of the ring, $E(k)$, for a continuum of values $k \in [0, 2\pi)$ (solid line, right axis), together with a semi-log plot of the momentum space distribution $|\psi_k|^2 = |\langle\psi_k|\psi(t)\rangle|^2$ of two wave packets in the bulk of the ring for $n = 100$ and $n = 1000$ (filled circles, left axis). The wave packets are guaranteed to be in the bulk by choosing the time $t$ as half the expected time taken by the wave packet to travel along the ring, $2gt = n/2$. The momentum space distribution is centered around $k_0 = \pi/2$, indicating a strong concentration of the wave packet around the linear part of the dispersion. For larger values of $n$, this distribution becomes narrower.

where $k_\ell = 2\pi\ell/n$ is the discrete lattice momentum for $\ell = 0, \ldots, n-1$. The momentum eigenstates $|\psi_{k_\ell}\rangle$ from (C18) diagonalize $H_{\text{bulk}}$ with dispersion relation (eigenvalues) given by $E(k_\ell) = 2g\cos(k_\ell)$. The coefficient of the wave packet $|\psi(t)\rangle$ in momentum space $\psi_{k_\ell}(t) = \langle\psi_{k_\ell}|\psi(t)\rangle$ are

$$\psi_{k_\ell}(t) = \frac{1}{\sqrt{n}} \sum_{j=0}^{n-1} e^{ik_\ell j} \psi_j(t). \tag{C19}$$

While the continuum solution in Sec. C 1 was used to capture the rapid preparation process of the wave packet, it falls short to capture dispersive effects which occur when traveling for longer times. To get a better grasp on this let us evolve the wave function up to some time $t_0$ which was chosen such that the wave packet has just left the initial ramp, $2gt_0 \gtrsim \lambda_\ell$. For times $t \geq t_0$, the evolution is well described in terms of the effective bulk Hamiltonian (C17). Numerically (see Fig. 2), we see that $\psi_{k_\ell}(t_0)$ is super-exponentially concentrated around $k_0 = \pi/2$ in momentum space, and therefore we can write the evolution for times later than $t_0$ as

$$\psi_j(t_0 + t) = \frac{1}{\sqrt{n}} \sum_{\ell=0}^{n-1} \psi_{k_0+q_\ell}(t_0) e^{-2igtE(k_0+q_\ell)} e^{-i(k_0+q_\ell)j}, \tag{C20}$$

where we set $k_\ell = k_0 + q_\ell$. The phase profile $e^{i\pi j/2} = i^j$ is the same we encountered in eq. (C4) and corresponds to the rapidly oscillating phase profile on top of a slowly varying wave function. Since we will later only be interested in absolute squares of the wave function this will not contribute and we will drop it below. Due to the strong concentration of $\psi_{k_\ell}(t_0)$ only the parts of the dispersion relation close to $k_0$ will contribute to the dy-

namics and we expand

$$E(k_0+q) = -2gq + \frac{2gq^3}{3!} + O(gq^5), \tag{C21}$$

allowing us to rewrite (C20) as

$$\psi_j(t_0+t) = \frac{1}{\sqrt{n}} \sum_{\ell=0}^{n-1} \psi_{k_0+q_\ell}(t_0) e^{-iq_\ell(j-2gt)} e^{-\frac{i}{3}gtq_\ell^3}, \tag{C22}$$

where we did not write the terms of order $O(q_\ell^5)$ and higher due to the strong concentration of $\psi_{k_\ell}(t_0)$ around $k_0$. The term with first order is responsible for translating the wave packet to the right with group velocity $2g$. Broadening of the wave packet is caused by the third-order term. Its consequences are the main concern of this section.

To assess how exactly the wave function, prepared by the initial ramp, traverses the bulk part of the ring clock we need to know the shape of the wave function at $t_0$. An exact treatment may be cumbersome analytically and give little additional insight. For our aim of determining the clock's ticking statistics, the scaling behavior from the hydrodynamic solution obtained in the continuum, turns out to be sufficient,

$$\psi_j(t) = \frac{1}{\sqrt{\lambda_\ell}} \Psi(t, x_j) \sim \frac{1}{\sqrt{\lambda_\ell}} f\left(\frac{j-2gt}{\lambda_\ell}\right), \tag{C23}$$

where we have set $x_j = j/\lambda_\ell$ in eq. (C16).

We will now consider the wave function of eq. (C23) in momentum space with respect to the discrete lattice momenta which gives us

$$\psi_{k_0+q_\ell}(t_0) = \frac{1}{\sqrt{n}} \sum_{j=0}^{n-1} e^{i(k_0+q_\ell)j} \psi_j(t_0) \tag{C24}$$

$$\sim \sqrt{\frac{\lambda_\ell}{n}} \int \mathrm{d}x e^{iq_\ell \lambda_\ell x} f\left(x - \frac{2gt_0}{\lambda_\ell}\right) \tag{C25}$$

$$= \sqrt{\frac{\lambda_\ell}{n}} \hat{f}(q_\ell \lambda_\ell), \tag{C26}$$

up to an irrelevant phase-shift due to the initial translation $2gt_0/\lambda_\ell$ in the final line. We replaced the Riemann sum $\sum_j = \lambda_\ell \sum_x \Delta x \to \int \mathrm{d}x$ with $\Delta x = 1/\lambda_\ell$ by an integral in the second line in the limit $\lambda_\ell \gg 1$. While we are not privy to the exact form of $f$, it is still possible to formally obtain its Fourier transformation $\hat{f}$, which is also strongly localized. From the dependence of the $\hat{f}$ on the length of the ramp $\lambda_\ell$ we conclude that the longer the initial ramp the stronger the localization in momentum space as found in Fig. 2.

Now that we have some notion of a state after the initial ramp, we are in the position to study the propagation of the wave packet using eq. (C22). In the limit of large $n \gg 1$, the discrete sum over $k_\ell \in \frac{2\pi}{n}\mathbb{Z}_n$ becomes an integral over $k \in [0, 2\pi)$ from which we obtain

$$\begin{aligned}\psi_j(t_0+t) &= \frac{\sqrt{\lambda_\ell}}{n} \sum_{\ell=0}^{n-1} \hat{f}(q_\ell\lambda_\ell) e^{-iq_\ell\lambda_\ell \frac{j-2gt}{\lambda_\ell}} e^{-i\frac{2gt}{\lambda_\ell^3}\frac{(q_\ell\lambda_\ell)^3}{3!}} \\ &\sim \sqrt{\lambda_\ell} \int_{-\infty}^{\infty} \frac{\mathrm{d}Q}{2\pi} \hat{f}(Q) e^{-iQ\frac{j-2gt}{\lambda_\ell}} e^{-i\frac{2gt}{\lambda_\ell^3}\frac{Q^3}{3!}},\end{aligned} \tag{C27}$$

Note that the length of an $k$-space interval is $\Delta k = 2\pi/n$ so the sum can be written as $\frac{1}{\Delta k}\sum_j \Delta k$ as a Riemann sum. In the limit $n \gg 1$ we replace the sum by an integral and obtain $\frac{2\pi}{n}\sum_{j=0}^{n-1} \to \int_{-\pi/2}^{3\pi/2} \mathrm{d}q$, where we have again used the translational invariance of the dispersion relation. Finally, we rescale the momentum $Q = q\lambda_\ell$ and since the $\hat{f}$ is strongly localized im momentum space, extending the limits of the integral to infinity will not incur too much of an error.

We now analyze the broadening of the wave packet due to the cubic term in the exponent in eq. (C27). Up to numerical constants the prefactor scales like $2gt/\lambda_\ell^3$ and therefore the longer the wave propagates the more relevant become the dispersive effects. This very observation is the main result of this section and crucial to finding the length of the ramp $\lambda_\ell$ which guarantees propagation with the least amount of spreading.

### 3. Emission

In the following two subsections, we first discuss in Sec. C 3 a the physics of the tick emission, investigating how the choice of couplings on the right ramp of Fig. 1(d) in the main text allows for the excitation to be emitted with unit probability once it arrives at the final site. Then, in the following Sec. C 3 b, we analyze the probability distribution of when the excitation is emitted based on the coherent evolution in the bulk of the ring clock, which allows us to obtain the scaling relations $\mathrm{E}[T] \sim n$ and $\mathrm{Var}[T] \sim n^{2/3}$.

#### *a. Apodization of the right ramp*

The choice of the couplings $g_j$ at the end of the ring cycle can be understood as a boundary matching problem, separate from the initialization of the wave packet at the beginning of the evolution. By maximizing for the clock precision $\mathcal{N}_\infty$, we find that the last few couplings $g_{n-2}, g_{n-3}, g_{n-4}, \ldots$ are larger than the coupling $g$ in the bulk of the ring, as visualized in Fig. 1(d) of the main text. The increase in the couplings is reminiscent of the optimal values used in the apodization of resonant tunneling structures to maximize transmission in a transport setting [15–17]. To have a unit transmission coefficient, it has been shown that only a small constant number of couplings before the emitting site have to be adjusted,

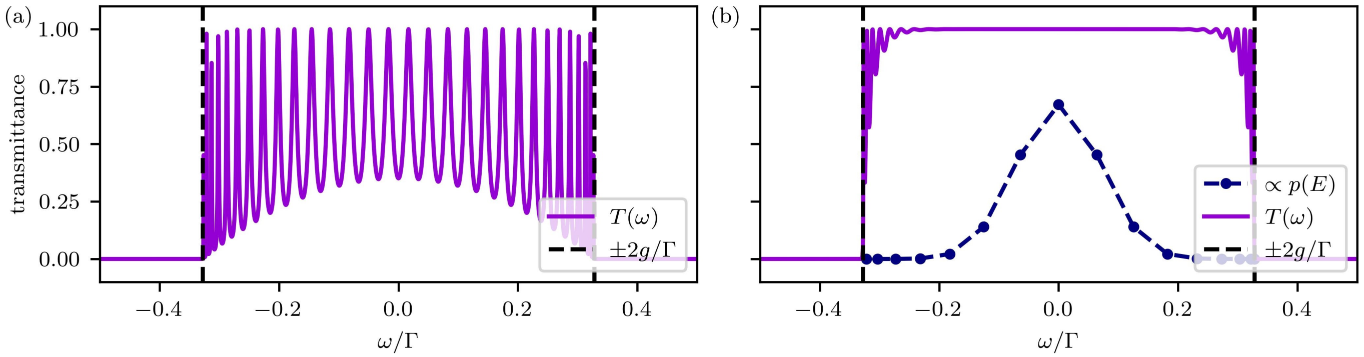


FIG. 3. Here, we show the transmission function of our setup. In (a) with flat couplings, and in (b) with apodized couplings, both for $n = 32$ sites. The transmission function $T(\omega)$ can be calculated using the expression in eq. (C28) by understanding the ring setup as a symmetric scatterer. In (a) we show the transmission function in the case where all nearest neighbor couplings $g_j = g$ are constant. The Fabry-Perot interference fringes prevent a unit probability emission of any spatially located travelling wave packet, due to the non-unity transmission function [15]. (b) Here, we have the transmission probability for the case where the couplings at the both ends of the scatterer are symmetrically apodized with a ramp as in the ring clock. Furthermore, we plot the energy probability distribution of the wave packet in the bulk of the ring, rescaled for better readability. We see that this setup has an approximately unit transmittance in the energy range where the wave packet has support. In both panels, the vertical dashed lines show the ends of the transmission spectrum given by the dispersion relation $E = 2g\cos(k) \in [-2g, 2g]$.

irrespective of how large the bulk is. Such an apodization of the final couplings prevents the reflection of most travelling waves within a certain energy band, and as it turns out, our maximization of clock precision leads to a choice of couplings that favor transmission over reflection (see comparison Fig. 3(a,b)).

*Transmission probability.* To make a more quantitative connection between tick emission in the ring clock and transimission in a quantum transport setting, one may wish to think of a chain instead of a ring. Since for the problem of emission only the final sites are relevant, the initialization ramp discussed in Sec. C 1 can be ignored. For quantifying the transmission behavior of the right ramp, we look at a symmetric setting, where the couplings on the left are the same as those on the right, that is, $g_j^{\text{sym.}} = g_{n-2-j}^{\text{sym.}}$. With our model as given in eq. (6), the couplings of the symmetrized setting are then described by $g_j^{\text{sym.}} = g + \mu_r \left(e^{-j/\lambda_r} + e^{-(n-j-1)/\lambda_r}\right)$. This setup allows for probing the emission ramp on the right independent of the details of wave packet preparation. Combining the resulting transmission function with the results on the travelling wave packet from our previous analysis in Sec. C 2, we find that the excitation lies precisely within the energy spectrum that our choice of couplings transmits, as shown in Fig. 3(b). Transmittivity can be quantified using the non-equilibrium Green's function (NEGF) method, for example, and in the following derivation we follow Ref. [18]. The transmission probability for a travelling wave of energy $\omega$ in our setting is given by

$$T(\omega) = \Gamma^2 \left|\left\langle n-1 \left| (\omega - H_{\text{eff}})^{-1} \right| 0 \right\rangle\right|^2 , \qquad \text{(C28)}$$

where the effective Hamiltonian is defined as in Sec. B 1, but with the symmetrized couplings, i.e.,

$$\begin{aligned} H_{\text{eff}} = &\sum_{j=0}^{n-2} g_j^{\text{sym.}} \, |j+1\rangle\langle j| + \text{h.c.} \\ &- i\frac{\Gamma}{2}\,|n-1\rangle\langle n-1| - i\frac{\Gamma}{2}\,|0\rangle\langle 0| \,. \end{aligned} \qquad \text{(C29)}$$

We show a comparison between how the transmission function looks like for different choices of couplings. In Fig. 3(a) we show the case without apodization by using flat couplings on the entire chain. In Fig. 3(b) we show the transmission function for the case where the ends of the chain are apodized using the optimal couplings from the ring clock. Even though we optimize for clock precision and not for transmittivity directly, this analysis shows that the optimal choice of couplings produces a travelling wave packet in the bulk of the ring whose energy distribution lies exactly within the transmission window of the apodized region at the end of the ring.

*The non-equilibrium Green's function.* The NEGF method is an exact, non-perturbative method to determine scattering amplitudes and for the following derivation we adapt the techniques from [18] to our context. In our setting it is sufficient to look at a system comprising three parts, a left port, the central region (the chain), and the right port. The left and right ports are microscopic models for the baths from which excitations enter and leave the chain. For the NEGF method, we restrict to the one-particle subspace $\mathcal{H}_L^1 \oplus \mathcal{H}_C^1 \oplus \mathcal{H}_R^1 \subseteq \mathcal{H}_L \otimes \mathcal{H}_C \otimes \mathcal{H}_R$ in the full Hilbert-space, as in the main text. As a consequence, we can write the Hamiltonian describing the

dynamics using

$$H = \begin{bmatrix} H_{LL} & H_{LC} & 0 \\ H_{CL} & H_{CC} & H_{CR} \\ 0 & H_{RC} & H_{RR} \end{bmatrix}. \tag{C30}$$

The Green's function must, by definition, satisfy the condition $(\omega \pm i0 - H)G(\omega)^{\pm} = \mathbb{1}$, where the notation $\pm i0$ can be understood as $\pm i\varepsilon$ in the limit of $\varepsilon \to 0^+$ in a distribution sense, to regularize the Green's function. In the following, we only work with the retarded Green's function, that is, the limit $+i0$ to ensure causality (see e.g. Chapter 8 from [18]), and we will drop the additional superscript and simply write $G(\omega)$ to mean $G(\omega)^+$. It will be useful to consider the following examples: the Green's function of the isolated leads are given by $g_L(\omega) = (\omega + i0 - H)^{-1}$ for the left lead and similarly for the right lead. Furthermore, the central element of the total Green's function for $H$ as defined in (C30) is the inverse of the effective Hamiltonian, $(\omega - H_{\text{eff}})\, G_{CC}(\omega) = \mathbb{1}$, where the effective Hamiltonian in this general setting is given by

$$H_{\text{eff}}(\omega) = H_{CC} + H_{CR} g_R(\omega) H_{RC} + H_{CL} g_L(\omega) H_{LC}. \tag{C31}$$

This can be derived by solving the three equations in the middle column of $(\omega + i0 - H)G(\omega) = \mathbb{1}$ for $G_{CC}(\omega)$. The self-energies $\Sigma_L(\omega) = H_{CL} g_L(\omega) H_{LC}$ (similarly for $R$) arise from the coupling of the central region to the leads and they can be split into a real part (the Lamb-shift) and an imaginary part, which is the non-Hermitian term describing the loss or gain of excitations due to the coupling of the central region to the ports. Generally, the imaginary term is identified as the sink term modeling particle loss (or gain) due to the coupling with the port, $\mathfrak{Im}[\Sigma_L(\omega)] \equiv \Gamma_L(\omega)/2$. By using the Shokhotski-Plemelj formula [19, 20] stating $(x \pm i0)^{-1} = \mathcal{P}x^{-1} \mp i\pi\delta(x)$ with $\mathcal{P}$ denoting the Cauchy-principle value, we can write

$$\Gamma_L(\omega) = -2\pi H_{CL} \delta(\omega - H_{LL}) H_{LC} \tag{C32}$$
$$= -i H_{CL} (g_L(\omega) - g_L(\omega)^\dagger) H_{LC} \tag{C33}$$

While the details of this expression are irrelevant to our purpose, one could in principle use them to exactly solve the scattering problem at hand. We, however, work in an approximate limit, where the imaginary term of the self-energies of the left and right port simplify to the expressions we have been using so far, where for the energy range $\omega$ of interest, we assume that

$$\mathfrak{Im}[\Sigma_L(\omega)] \equiv \frac{\Gamma_L(\omega)}{2} = -\frac{\Gamma}{2} |0\rangle\langle 0|_C\,, \tag{C34}$$

and,

$$\mathfrak{Im}[\Sigma_R(\omega)] \equiv \frac{\Gamma_R(\omega)}{2} = -\frac{\Gamma}{2} |n-1\rangle\langle n-1|_C\,. \tag{C35}$$

Such a model can be recovered using a Breit-Wigner – or Markov – approximation [21], as already implicitly done in the main text by using the Lindblad master equation. By furthermore absorbing the real part of the self-energy in the Hamiltonian $H_{CC}$ and shifting the diagonal of the chain Hamiltonian to 0, we recover the expression in eq. (C29) that we have already been using in the main part of the paper (up to the symmetrized setting).

*Derivation of the transmission probability.* Given the consistency with the previous model, i.e., equality of $H_{\text{eff}}$ as in (C29) and as derived using the NEGF method (C31), we are ready to move further towards deriving the transmission probability $T(E)$ from eq. (C28). To this end, we look at the eigenvalue equation of the full Hamiltonian including both ports and the chain region,

$$(\omega - H)\,|\Psi\rangle = 0, \text{ where } |\Psi\rangle := \begin{bmatrix} |\phi_L\rangle + |\chi_L\rangle \\ |\psi_C\rangle \\ |\chi_R\rangle \end{bmatrix}. \tag{C36}$$

Physically, we split the wave function on the left port into one incoming contribution $|\phi_L\rangle$ and a back-scattered contribution $|\chi_L\rangle$. Furthermore, we have the wave function $|\psi_C\rangle$ in the chain and the transmitted function $|\chi_R\rangle$ in the right port. Given the incoming wave function $|\phi_L\rangle$ as the independent variable, we want to determine the responses $|\chi_L\rangle, |\chi_R\rangle$ and $|\psi_C\rangle$. Note that none of these terms is necessarily normalized, we only chose $|\phi_L\rangle$ to be normalized by convention. Furthermore, we assume $(\omega - H_{LL})\,|\phi_L\rangle = 0$, that is, we chose the incoming wave to have energy $\omega$, then, we can solve the time-independent Schrödinger equation (C36) for $|\chi_L\rangle$ and $|\chi_R\rangle$. We present the examplary case for $\chi_L$, by looking at the first row,

$$(\omega - H_{LL})(|\phi_L\rangle + |\chi_L\rangle) - H_{LC}\,|\psi_C\rangle = 0, \tag{C37}$$

from which it follows by using $(\omega - H_{LL})\,|\phi_L\rangle = 0$ that $(\omega - H_{LL})\,|\chi_L\rangle = H_{LC}\,|\psi_C\rangle$. Formally, we can only invert this equation by adding a regularizing imaginary part to the energy $\omega \mapsto \omega + i0$, which then allows us to invert this equation to give $|\chi_L\rangle = g_L(\omega) H_{LC}\,|\psi_C\rangle$. In the literature, as e.g. Ref. [18], this problem is usually solved by adding an additional source-term $|S\rangle$ to the time-independent Schrödinger equation $(\omega + i\varepsilon - H)\,|\Psi\rangle = |S\rangle$, which then vanishes in the limit $\varepsilon \to 0$. For the right response we can similarly write $|\chi_R\rangle = g_R(\omega) H_{RC}\,|\psi_C\rangle$ using the last row of eq. (C36). Inserting this into the middle row gives us an expression for $|\psi_C\rangle$ as a function of $|\phi_L\rangle$,

$$(\omega - H_{\text{eff}})\,|\psi_C\rangle = H_{CL}\,|\phi_L\rangle\,. \tag{C38}$$

Using the same regularization from before $\omega \to \omega + i0$ allows us to formally solve for the wave function in the chain $|\psi_C\rangle = G_{CC}(\omega) H_{CL}\,|\phi_L\rangle$, giving us the expression for the scattered wave-function in the right port as a function of the incoming wave $|\chi_R\rangle = g_R(\omega) H_{RC} G_{CC}(\omega) H_{CL}\,|\phi_L\rangle$. Generally, there are many possible left-port solutions $|\phi_L^k\rangle$ for the incoming wave, and for later use, we will denote these states with the superscript $k$. Given the relationship between $|\chi_R\rangle$ and

$|\phi_L^k\rangle$, we can determine the probability current from the left port to the right port using the time-dependent Schrödinger equation $i\partial_t |\Psi\rangle = H|\Psi\rangle$, noting that $|\Psi\rangle$ is still the solution of the stationary equation (C36). We are interested in the probability current into the right port, for which we need to calculate $i_T^k = \partial_t \langle \chi_R|\chi_R\rangle$, with the subscript $T$ for the transmitted current, and the superscript $k$ for indexing the implicit incoming state $|\phi_L^k\rangle$. One may think that because $|\Psi\rangle$ is the solution to the stationary Schrödinger equation, the probability current is zero; here, this is not the case, because we look at the perturbed equation where $\omega \mapsto \omega + i0$, thus giving us the non-zero current,

$$i_T^k = -i\langle \chi_R| (H_{RC}|\psi_C\rangle + H_{RR}|\chi_R\rangle) + \text{h.c.} \tag{C39}$$
$$= -i\left(\langle \chi_R|H_{RC}|\psi_C\rangle - \langle \psi_C|H_{CR}|\chi_R\rangle\right) \tag{C40}$$

Using the previous result that $|\chi_R\rangle = g_R(\omega)H_{RC}|\psi_C\rangle$ together with the result that the imaginary part of the self energy equals the non-Hermitian loss term $\mathfrak{Im}[\Sigma_R(\omega)] = \Gamma_R(\omega)$, we can simplify

$$i_T^k = i\langle \psi_C|H_{CR}\left(g_R(\omega) - g_R(\omega)^\dagger\right)H_{RC}|\psi_C\rangle \tag{C41}$$
$$= -\langle \psi_C|\Gamma_R|\psi_C\rangle\,, \tag{C42}$$

where we dropped the $\omega$ argument in the last line for readability. We will from now on not explicitly write the $\omega$ argument anymore in the cases where it is unambiguous. By using the relationship between $|\psi_C\rangle$ and the incoming wave $|\phi_L^k\rangle$ derived in eq. (C38), we can write the current as a function of the incoming wave $|\phi_L\rangle$. The transmitted current expressed as a function of the incoming wave $|\phi_L^k\rangle$ is thus given by,

$$i_T^k = -\left\langle \phi_L^k \middle| H_{LC}G_{CC}^\dagger \Gamma_R G_{CC} H_{CL} |\phi_L^k\right\rangle \tag{C43}$$
$$= -\operatorname{tr}\left[H_{CL}|\phi_L^k\rangle\!\langle\phi_L^k| H_{LC}G_{CC}^\dagger\Gamma_R G_{CC}\right]. \tag{C44}$$

Summing over all possible eigenstates $|\phi_L^k\rangle$ with energy $\omega_{L,k} = \omega$ and occupation probability $p(\omega_k)$ we obtain the total transmitted probability current,

$$i_T = \sum_k p(\omega_k) i_T^k \tag{C45}$$
$$= \int d\omega p(\omega) \sum_k \delta(\omega - \omega_{L,k}) i_T^k \tag{C46}$$
$$= \int \frac{d\omega}{2\pi} p(\omega) \operatorname{tr}\left[\Gamma_L G_{CC}^\dagger \Gamma_R G_{CC}\right], \tag{C47}$$

from which we can extract the transmission probability

$$T(\omega) = \operatorname{tr}\left[\Gamma_L G_{CC}^\dagger \Gamma_R G_{CC}\right] \tag{C48}$$

as in [18]. Inserting the operators from our model (C34) and (C35), $\Gamma_L = \Gamma|0\rangle\!\langle 0|$ and $\Gamma_R = \Gamma|n-1\rangle\!\langle n-1|$, we recover the expression $T(\omega) = \Gamma^2|\langle 0|G_{CC}(\omega)|n-1\rangle|^2$ from eq. (C28) at the beginning of this section.

#### b. *Scaling of clock precision with ring length*

Here, we combine all the previous results to obtain the theoretical result displayed in eq. (7) of the main text. This result relies on the PDF of the ticks obtained in Sec. B 1, the preparation by the initial ramp from Sec. C 1, the subsequent propagation through the bulk of the ring from Sec. C 2 and eventually the emission or tick event was discussed in Sec. C 3.

Our starting point is the PDF in eq. (B7) and for the following analysis it will be beneficial to decompose the effective Hamiltonian from eq. (B5) in two parts,

$$H_{\text{eff}} = \underbrace{\sum_{j=0}^{n-2}\left(-\mu_\ell e^{-j/\lambda_\ell} + g\right)\left(|j\rangle\!\langle j+1| + \text{h.c.}\right)}_{=H_0} + \underbrace{\sum_{j=0}^{n-2}\mu_r e^{(j-(n-1))/\lambda_r}\left(|j\rangle\!\langle j+1| + \text{h.c.}\right) - \frac{i}{2}J^\dagger J}_{=K} \tag{C49}$$

where $H_0$ describes the dynamics of preparation by the ramp and bulk propagation (see Secs. C 1 and C 2). We refer to this as the free part of the evolution. The non-hermitian Hamiltonian $K$ corresponds to dynamics when the wave packet hits the final apodization region and its subsequent conversion to a tick of the clock, we refer to this as the interaction part. The evolution generated by the effective Hamiltonian $H_{\text{eff}}$ expanded in a Dyson series reads

$$e^{-iH_{\text{eff}}t} = \underbrace{e^{-iH_0 t}}_{=U(t)} + \underbrace{e^{-iH_0 t}\sum_{k=1}^{\infty}(-i)^k \int_0^t \mathrm{d}\tau_1 \int_0^{\tau_1}\mathrm{d}\tau_2 \cdots \int_0^{\tau_{k-1}} \mathrm{d}\tau_k\, K(\tau_1)\cdots K(\tau_k)}_{=\mathcal{V}(t)}, \tag{C50}$$

where we defined Hamiltonian in the interaction picture $K(t) = e^{iH_0 t}Ke^{-iH_0 t}$. With the decomposition of the

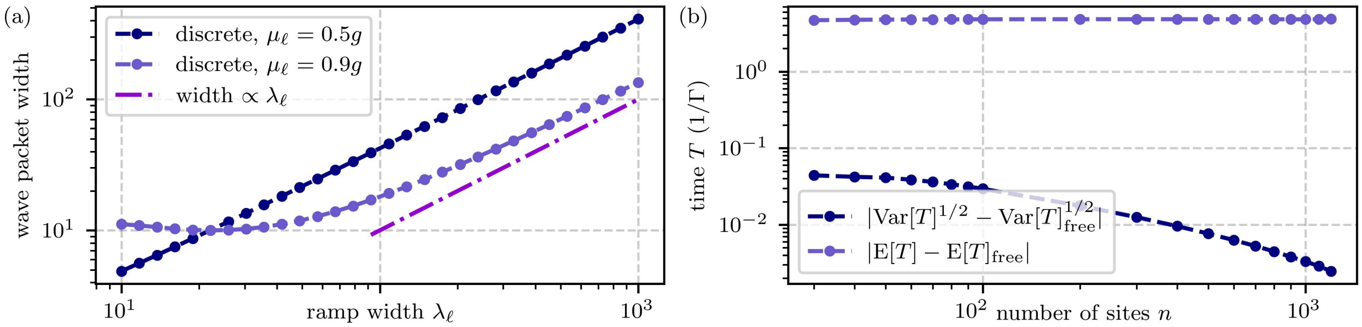


FIG. 4. In (a), we show how the width of the wave packet scales with the length $\lambda_\ell$ of the initialization ramp. The continuum limit predicts a linear scaling of the width as shown in (C23) in the limit of large values of $\lambda_\ell$. This plot confirms that the scaling holds for large values of $\lambda_\ell$ regardless of the choice of the parameter $\mu_\ell$ which only affects how large $\lambda_\ell$ must be for the continuum limit to dominate also in the discrete case. In (b), we show a comparison between the numerically exact tick probability density $P[T=t]$ and the approximation from eq. (C51) where the lossy contribution is neglected. We are looking at the difference between the exact average value $\mathrm{E}[T]$ and the one obtained from the approximation $\mathrm{E}[T]_{\mathrm{free}}$ of the free theory. Similarly, we also plot the difference between the standard deviations $\mathrm{Var}[T]^{1/2}$ obtained using the two different methods. We find that the error we obtain with this approximation stays constant for the average values and even shrinks for the standard deviation, justifying the approximation made in Sec. C 3 by neglecting the lossy contributions to the tick PDF.

effective time evolution in eq. (C50), the tick probability can also be decomposed into two parts,

$$P[T=t] = \underbrace{\Gamma|c_0(t)|^2}_{=p_0(t)} + p_1(t), \tag{C51}$$

where

$$p_1(t) = \Gamma\left(|c_1(t)|^2 + 2\mathfrak{Re}\left[c_0(t)^* c_1(t)\right]\right). \tag{C52}$$

The coefficients $c_0(t)$ and $c_1(t)$ are obtained from the decomposition of $e^{-iH_{\mathrm{eff}}t}$ into the free and interacting part,

$$c_0(t) = \langle n-1|U(t)|0\rangle\,, \quad c_1(t) = \langle n-1|\mathcal{V}(t)|0\rangle\,. \tag{C53}$$

This shows that the tick PDF is made up of two contributions; one exclusively attributed to propagation and the other is the correction coming from apodization as well as emission.

The main result of Sec. C 3 a is that the couplings $g_j$ as optimized by eq. (B11) match the right ramp to the tick rate $\Gamma$. As shown in Fig. 3(a,b), the result is that the right ramp transmits the wave packet with close to unit probability without reflection. Consequently, the tick PDF is, up to small corrections, essentially dominated by the bulk propagation, i.e., the free term $p_0(t)$. This means that we may neglect the lossy interaction contribution $p_1(t)$ without incurring on a significant error for the discussion below. In Fig. 4(b) a numerical analysis illustrates how much the predicted first and second moments of the tick time differ when using only the free part $p_0(t)$ instead of the full theory $P[T=t]$.

We now use the expression for the propagating wave function in the continuum limit from eq. (C27) and evaluate $c_0(t)$ at time close to the time $n/2g$ where the wave packet has reached the right ramp. For this, we identify $c_0(t) = \psi_{n-1}(t)$, and we recall that we chose an offset time $t_0$ for obtaining eq. (C27) such that $n \gg 2gt_0 \gtrsim \lambda_\ell$, i.e., the wave packet has just left the left ramp but is still far away from the end of the ring. Then, we can write $c_0(t) = c_0(t_0 + (t-t_0)) \sim c_0(t_0 + t)$ because we are considering times $t \sim n/2g$ much larger than $t_0$. Looking at small deviations $t = n/2g + \delta t$, we can write

$$c_0\left(t\right) \sim \sqrt{\lambda_\ell}\int_{-\infty}^{\infty} \frac{\mathrm{d}Q}{2\pi}\hat{f}(Q)e^{+iQ\frac{2g\delta t}{\lambda_\ell}}e^{-i\frac{n}{\lambda_\ell^3}\frac{Q^3}{3!}}, \tag{C54}$$

where we dropped the $\delta t \ll n/2g$ term in the $Q^3$ expression. Note, that the prefactor of the third order term in the exponent scales like $n/\lambda_\ell^3 Q^3$, which is responsible for the strength of the broadening of the wave packet after traversing the ring. The skewing of the wave packet thus proliferates with increasing ring size $n$. However, this expression suggests that choosing the appropriate scaling of $\lambda_\ell \sim n^{1/3}$ with the system size, this effect can be mitigated. This means that the error from the cubic term can be bounded from above by an arbitrarily small constant, and the wave packet thus does not skew as it propagates along the ring. As discussed in the main text, we find

$$\lambda_\ell \overset{\text{num.}}{\sim} n^{0.35} \overset{\text{th.}}{\sim} n^{1/3}, \tag{C55}$$

which suggests that the scaling found in the numerical optimization is in good agreement with the necessary scaling predicted by our theory. Integrating the expression in eq. (C54), the cubic term can thus be dropped if the asymptotic constant for $n^{1/3}/\lambda_\ell = \varepsilon$ is sufficiently small, and we recover the expression from eq. (C23) for the absolute square or the tick PDF,

$$P[T=t] \sim \Gamma|c_0(t)|^2 \sim \frac{\Gamma}{n^{1/3}} h\left(\frac{n-2gt}{n^{1/3}}\right)^2, \tag{C56}$$

where

$$h(x) = \int_{-\infty}^{\infty} \frac{\mathrm{d}Q}{2\pi} \hat{f}(Q) e^{-iQx - i\varepsilon \frac{Q^3}{3!}} \sim f(x)^2. \tag{C57}$$

Note, here, we have reinstated the absolute time $t$ again. The prefactor $n^{-1/3}$ comes from the correct normalization, as also present in eq. (C23). Due to the strong concentration of $\hat{f}$ we expect that so is $h$ and that $\int \mathrm{d}x\, x h(x) < \infty$ as well as $\int \mathrm{d}x\, x^2 h(x) < \infty$ are finite. Therefore, we find for the first moment of the tick PDF the scaling

$$\mathrm{E}[T] \sim \frac{\Gamma}{n^{1/3}} \int_{-\infty}^{\infty} \mathrm{d}t\, t\, h\left(\frac{n-2gt}{n^{1/3}}\right)^2 \sim n, \qquad n \to \infty. \tag{C58}$$

For the second moment, i.e., the variance we obtain the scaling

$$\mathrm{Var}[T] \sim \frac{1}{n^{1/3}} \int_{-\infty}^{\infty} \mathrm{d}t\, (t - \mathrm{E}[T])^2 h\left(\frac{n-2gt}{n^{1/3}}\right)^2 \tag{C59}$$

$$\sim n^{2/3} \int_{-\infty}^{\infty} \mathrm{d}x\, x^2 h(x)^2 \tag{C60}$$

$$\sim n^{2/3}, \qquad n \to \infty. \tag{C61}$$

This scaling is significantly below $\mathrm{Var}[T] \sim n^2$ which we expect from ballistic transport. Together with eq. (5) we obtain eq. (7) which is the main result of Sec. C. In the following section, we give an explanation for how the loss term in eq. (C51) can be neglected due to the boundary matching of the right ramp.

## Appendix D: Exponential scaling of precision with entropy production

We now switch back to the regime where the jump process $\overline{J}$ participates in the evolution. In the following, we discuss how one can calculate the clock precision as defined in the main text eq. (4),

$$\mathcal{N}_\Sigma = \lim_{t\to\infty} \frac{\mathrm{E}[N(t)]}{\mathrm{Var}[N(t)]}, \tag{D1}$$

given the generators of the evolution for the ring clock and how $\mathcal{N}_\infty$ and $\mathcal{N}_\Sigma$ are related in the infinite entropy production regime (Sec. D 1). Subsequently, we examine what happens in detail if the assumption on the infinite entropy production is dropped. Our goal is to quantify how much $\mathcal{N}_\Sigma$ and $\mathcal{N}_\infty$ then differ as a function of the finite entropy production (Sec. D 2).

### 1. Calculating clock precision in the reversible regime

Formally, the number of ticks $N(t)$ are defined as an integrated stochastic current $N(t) = \int_0^t \mathrm{d}N(\tau)$. There, the jumps $|n-1\rangle \to |0\rangle$ ($|0\rangle \to |n-1\rangle$) lead to the incremental increase $\mathrm{d}N(t) = +1$ (decrease $\mathrm{d}N(t) = -1$) respectively. We can resolve the master equation evolution of the ring clock's state $\rho(t)$ with respect to the number of times the clock has ticked by introducing a free counting field $\chi$ [1, 7],

$$\rho(t, \chi) = \sum_{k\in\mathbb{Z}} \rho^{(k)}(t) e^{ik\chi}. \tag{D2}$$

The state $\rho^{(k)}(t)$ is the non-normalized clock state given that the net number of ticks at time $t$ is $k$. The trace $\mathrm{tr}\left[\rho^{(k)}(t)\right] = P[N(t) = k]$ reveals the probability of this event. To obtain the form $\rho(t,\chi)$ from a dynamical time-evolution, we can introduce the tilted Liouvillian $\mathcal{L}(\chi)$ of the system as already introduced in the Methods,

$$\mathcal{L}(\chi) = \mathcal{L}_0 + e^{+i\chi}\mathcal{L}_+ + e^{-i\chi}\mathcal{L}_-. \tag{D3}$$

Here, the three parts of the Liouvillian are defined as $\mathcal{L}_+ \cdot\ = J \cdot J^\dagger$ for the jumps counted as positive ticks, and $\mathcal{L}_- \cdot\ = \overline{J} \cdot \overline{J}^\dagger$ for the jumps counted as negative ticks. Thus, the counting field has a positive sign for the forward jumps $e^{+i\chi}\mathcal{L}_+$ and a negative sign for the backwards jumps $e^{-i\chi}\mathcal{L}_-$. Finally, the term $\mathcal{L}_0$ generates the evolution conditioned on no jump ocurring, given by (as in eq. (9) from the Methods),

$$\mathcal{L}_0 \cdot\ = -i[H, \cdot\,] - \frac{1}{2}\left\{J^\dagger J, \cdot\right\} - \frac{1}{2}\left\{\overline{J}^\dagger \overline{J}, \cdot\right\}. \tag{D4}$$

The equation $\dot{\rho}(t,\chi) = \mathcal{L}(\chi)\rho(t,\chi)$ then generates the time-evolution resulting in the state $\rho(t,\chi)$ of the form as in eq. (D2). The cumulant generating function $C(\chi, t)$ for $N(t)$ can be obtained using the eigenvalue $\lambda(\chi)$ with the largest real part of the tilted Liouvillian $\mathcal{L}(\chi)$ [1]. In the long-time limit $t \to \infty$, it holds that $C(\chi, t) = t\lambda(\chi) + O(1)$, allowing us to determine the asymptotic values for $\mathrm{E}[N(t)]$ and $\mathrm{Var}[N(t)]$,

$$\lim_{t\to\infty} \frac{\mathrm{E}[N(t)]}{t} = -i\frac{\mathrm{d}}{\mathrm{d}\chi}\lambda(\chi)\Big|_{\chi=0}, \tag{D5}$$

and

$$\lim_{t\to\infty} \frac{\mathrm{Var}[N(t)]}{t} = -\frac{\mathrm{d}^2}{\mathrm{d}\chi^2}\lambda(\chi)\Big|_{\chi=0}, \tag{D6}$$

and therefore the clock precision as in (4). These expressions are general and they hold regardless of whether the reverse ticking process generated by $\overline{J}$ is present or not.

In Sec. B, we have calculated the clock precision in terms of the first passage time $T$, where $\overline{J} = 0$ was assumed. This would for example be realized in the limit of infinite entropy production per tick $\Sigma_{\text{tick}} = \infty$. If we formally take this limit, the counting variable $N(t)$ falls into the class of renewal-processes [22]. Renewal processes are generalized Poisson processes, where the time between successive events is independently and identically distributed (i.i.d.) according to some waiting time

distribution. For the Poisson process, the waiting time is exponentially distributed, in our case, it is distributed according to $P[T=t]$ as given by the formula (B7). The reason why the time between ticks is i.i.d. distributed, is that after every tick, the clock resets to the same initial state is $\rho_0 = |0\rangle\langle 0|$. Renewal process have particularly well-behaved properties such that the asymptotic moments of $N(t)$ are related to that of $T$ in the following way [22, 23],

$$\lim_{t\to\infty} \frac{\mathrm{E}[N(t)]}{t} = \frac{1}{\mathrm{E}[T]}, \tag{D7}$$

and

$$\lim_{t\to\infty} \frac{\mathrm{Var}[N(t)]}{t} = \frac{\mathrm{Var}[T]}{\mathrm{E}[T]^3}. \tag{D8}$$

In the case of formally infinite entropy production per tick, the clock precision defined with respect to $N(t)$ is therefore the same as the one defined with respect to $T$, i.e., we have

$$\mathcal{N}_\Sigma = \lim_{t\to\infty} \frac{\mathrm{E}[N(t)]}{\mathrm{Var}[N(t)]} \to \mathcal{N}_\infty = \frac{\mathrm{E}[T]^2}{\mathrm{Var}[T]}. \tag{D9}$$

### 2. Clock precision at finite entropy production

Having found a way to determine the moments of $N(t)$ and $T$ and relating them in the case where $\overline{J}=0$, the question is raised of how much is the equality (D9) violated if $\overline{J}$ enters as a small but non-zero perturbation. To deal with this issue we relax the limit $\Sigma_{\text{tick}} = \infty$ to some finite values of $\Sigma_{\text{tick}}$, and introduce the perturbative parameter $\delta = e^{-\Sigma_{\text{tick}}}$ to write $\overline{J} = \sqrt{\delta} J^\dagger$. We can thus re-express eq. (D3) as follows,

$$\mathcal{L}(\chi,\delta) = -i\,[H,\cdot] - \frac{1}{2}\left\{J^\dagger J,\cdot\right\} + e^{i\chi} J\cdot J^\dagger + \delta\left(-\frac{1}{2}\left\{JJ^\dagger,\cdot\right\} + e^{-i\chi} J^\dagger\cdot J\right). \tag{D10}$$

We recover the following two limiting cases:

- If $\delta = 0$, we get eqs. (D7), (D8) and (D9), with $\mathcal{N}_\Sigma \to \mathcal{N}_\infty$.
- If $\delta > 0$ is small, the reverse jump $\overline{J}$ enters as a perturbation $\mathcal{L}(\chi,\delta) = \mathcal{L}(\chi,0) + O(\delta)$. Since $\mathcal{N}_\Sigma$ is a continuous function of ($\chi$-derivatives) of the dominant eigenvalues of $\mathcal{L}(\chi,\delta)$, we anticipate that the $\delta$-perturbation of $\mathcal{L}$ also leads to a $\delta$-perturbation of the precision $\mathcal{N}_\Sigma = \mathcal{N}_\infty(1+O(\delta))$.

The goal of the following section is to make the second case rigorous in the sense that we determine the prefactors in the $O(\delta)$ perturbation. We want to end up with a $\delta$ that scales with the ring length as $\delta = n^{-\zeta}$ for some value of $\zeta > 0$, such that the correction $O(\delta) \to 0$ vanishes for large ring lengths $n \to \infty$. What we have not considered explicitly so far: the prefactor in the $O$-notation for the perturbation of the clock precision could also scale with $n$. For example, if the prefactor were to grow exponentially with $n$, $\delta = n^{-\zeta}$ would be insufficient to make the error vanish, and one would have to chose $\delta$ differently. For reasons detailed in the following analysis, the perturbation is well-behaved and the prefactor only scales polynomially. The clock precision is therefore perturbed as follows,

$$|\mathcal{N}_\infty - \mathcal{N}_\Sigma| = O\left(n^{c-\zeta}\right), \tag{D11}$$

now including all $n$-dependencies in the $O$-notation. In Fig. 5(a), we visualize this bound by plotting the absolute difference $|\mathcal{N}_\infty - \mathcal{N}_\Sigma|$ and in Fig. 5(b), we show how $\mathcal{N}_\Sigma$ scales if $\zeta$ is not chosen large enough. The constant $\zeta > 0$ comes from the choice of $\delta = n^{-\zeta}$ and $c > 0$ is a constant related to the spectral gap of the ring clock Liouvillian, which we will get to later. What this tells us is that there exists a choice of $\zeta > c$, where the error between $\mathcal{N}_\Sigma$ and $\mathcal{N}_\infty$ becomes negligible for large values of $n$. We find that $\zeta = 4$ is a choice that works, as shown in Fig. 5(a). Using the identification $\delta = e^{-\Sigma_{\text{tick}}}$ gives us logarithmically growing entropy production per tick $\Sigma_{\text{tick}} = 4\log n$. Combined together with the scaling $\mathcal{N}_\Sigma \sim n^{1.31}$ that we recover from that of $\mathcal{N}_\infty$, we find our desired result,

$$\mathcal{N}_\Sigma = e^{\Omega(\Sigma_{\text{tick}})}, \tag{D12}$$

clock precision and entropy per tick are exponentially separated.

*Detailed analysis.* To arrive at the expansion in eq. (D11), we first look at how $\mathrm{E}[N(t)]$ and $\mathrm{Var}[N(t)]$ behave for finite values of $\delta$. Here, we again assume $\delta$ to be an independent parameter and only in the end we will prescribe a relationship $\delta = n^{-\zeta}$. Formally, we can write the first two moments in terms of a power series in the perturbation $\delta$ as follows by using the results from eq. (D5),

$$\lim_{t\to\infty} \frac{\mathrm{d}}{\mathrm{d}t}\mathrm{E}[N(t)] = \underbrace{-i\lambda_{10}}_{=1/\mathrm{E}[T]} - i\sum_{k=1}^{\infty} \lambda_{1k}\delta^k, \tag{D13}$$

with the notation $\lambda_{jk} := \partial_\chi^j \partial_\delta^k \lambda(\chi)\big|_{\delta=\chi=0}$, and for later use $\lambda_j := \partial_\chi^j \lambda(\chi)\big|_{\chi=0}$. For the second moment, we have by using eq. (D6)

$$\lim_{t\to\infty} \frac{\mathrm{d}}{\mathrm{d}t}\mathrm{Var}[N(t)] = \underbrace{-\lambda_{20}}_{=\mathrm{Var}[T]/\mathrm{E}[T]^3} + \sum_{k=1}^{\infty} \lambda_{2k}\delta^k. \tag{D14}$$

The coefficients in the higher order expansion, $\lambda_{jk}$ are also implicitly functions of $n$, and to ensure that the corrections to eqs. (D13) and (D14), stemming from finite $\delta$, are small, these coefficients must not grow too quickly as a function of $n$. Let us thus examine in more detail

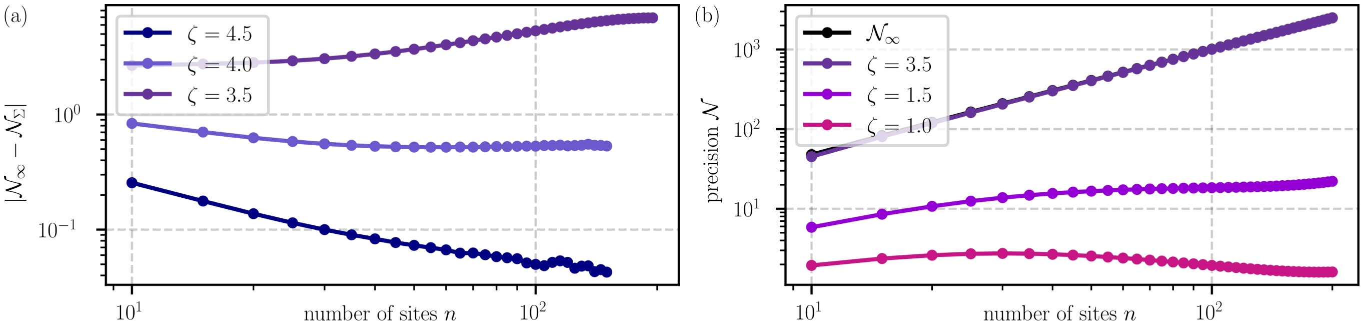


FIG. 5. Comparisons between the clock precision $\mathcal{N}_\Sigma$ in case of finite entropy-production and $\mathcal{N}_\infty$ in the limit $\delta = 0$ are shown. (a) We plot the difference $\mathcal{N}_\infty - \mathcal{N}_\Sigma$ for different choices of the exponent in $\delta = n^{-\zeta}$ in a loglog-scale. We see that for all three choices among $\zeta \in \{3.5, 4.0, 4.5\}$, the error of the clock precision is negligible on the scale of $\mathcal{N}_\Sigma$. (b) This is a loglog-plot of the clock precision $\mathcal{N}_\Sigma$ with a comparison to $\mathcal{N}_\infty$. We look at further choices of $\zeta \in \{1.0, 1.5, 3.5\}$ and we find that if $\zeta$ is chosen too small, the perturbation from the reverse tick process destructively affects the clock precision. For $\zeta = 3.5$, $\mathcal{N}_\Sigma$ and $\mathcal{N}_\infty$ overlap on the scale of this plot.

these coefficients $\lambda_{jk}$. They can be determined by looking at the eigenvalue problem $\mathcal{L}(\chi)\omega(\chi) = \lambda(\chi)\omega(\chi)$ written as a perturbation series in $\delta$ and $\chi$. The expansion with respect to $\chi$ gives us the derivatives of the dominant eigenvalue $\lambda(\chi)$ which equal the cumulants of $N(t)$ as detailed in eqs. (D5) and (D6). On the other side, the expansion in $\delta$ gives us the two expansion coefficients in (D13) and (D14). The eigenvalue problem can be explicitly written as

$$\left(\sum_{i,j\geq 0} \mathcal{L}_{ij}\chi^i\delta^j\right)\left(\sum_{i,j\geq 0} \omega_{ij}\chi^i\delta^j\right) = \left(\sum_{i,j\geq 0} \lambda_{ij}\chi^i\delta^j\right)\left(\sum_{i,j\geq 0} \omega_{ij}\chi^i\delta^j\right). \tag{D15}$$

We can solve this equation iteratively order by order in powers of $\chi$ and $\delta$. For concreteness, we are interested in expansions in $\chi$ up and including second order, i.e., $i \leq 2$, and for $\delta$, we are interested in the whole power series because we want to bound the overall errors entering eqs. (D13) and (D14). For the expansion coefficient of $\chi^i\delta^j$, the equation is given by the following expression,

$$\sum_{k+\ell=i}\sum_{m+n=j} \mathcal{L}_{km}\omega_{\ell n} = \sum_{k+\ell=i}\sum_{m+n=j} \lambda_{km}\omega_{\ell n}, \tag{D16}$$

where we note that $\sum_{k+\ell=i}$ is a shorthand for the sum over the set $\{k, \ell : k + m = i; k, m \in \{0, \ldots, i\}\}$ and by definition $\mathcal{L}_{km} = 0$ for indices $m > 1$. The recursive solution works as follows: we know that $\lambda_{00} = 0$ is the unique eigenvalue corresponding to the system's steady-state $\omega_{00}$ which we can determine initially. Then, we may assume that we know all the terms up to $\omega_{i-1,j}$ and $\lambda_{i-1,j}$ or $\omega_{i,j-1}$, and $\lambda_{i,j-1}$. In the next step, we can determine $\omega_{i,j}$ and $\omega_{i,j}$ as follows. First, we calculate

$$\lambda_{ij} = \mathrm{tr}\left[\sum_{k,\ell,m,n\in\Lambda_{ij}} (\mathcal{L}_{km}\omega_{\ell n} - \lambda_{km}\omega_{\ell n})\right], \tag{D17}$$

with the index set $\Lambda_{ij} = \{k, \ell, m, n : k + \ell = i,\ m + n = j,$ and $k, \ell \in \{0, \ldots, i\},\ m, n \in \{0, \ldots, j\}\}\backslash\{(0, i, 0, j)\}$. The terms $km = 00, \ell n = ij$ are not present because $\mathrm{tr}[\mathcal{L}_{00}\,\cdot\,] = 0$ and $\lambda_{00} = 0$. Therefore, on the right-hand side of eq. (D17), only terms of order lower than $ij$ enter, which, by assumption we have already computed. Next, we can obtain the state $\omega_{ij}$ as follows,

$$\omega_{ij} = \mathcal{L}_{00}^{+}\left(\sum_{k,\ell,m,n\in\Omega_{ij}} (\lambda_{km}\omega_{\ell n} - \mathcal{L}_{km}\omega_{\ell n})\right), \tag{D18}$$

where $\Omega_{ij} = \Lambda_{ij} \cup \{(0, i, 0, j)\}$. Note that $\lambda_{00} = 0$ and thus the expression $\lambda_{00}\omega_{ij}$ does not appear on the right-hand side of eq. (D18). The operator $\mathcal{L}_{00}^{+}$ is the Drazin inverse of $\mathcal{L}_{00}$ which inverts $\mathcal{L}_{00}$ except on the subspace of the 0 eigenvalue [7]. Steps (D17) and (D18) together with the base case of the induction, $\lambda_{00} = 0$ and $\omega_{00}$ being the steady-state, allow us to compute $\omega_{ij}$ and $\lambda_{ij}$ for arbitrary $i, j$.

From the functional form of $\lambda_{ij}$ and $\omega_{ij}$ we can conclude that $\lambda_{ij}$ is a linear affine function of $(\mathcal{L}_{00}^{+})^{i+j-1}, \ldots, \mathcal{L}_{00}^{+}$. This is an important observation because if we want to bound how fast $\lambda_{ij}$ grows with $n$, we need to know how quickly the largest eigenvalue of $\mathcal{L}_{00}^{+}$ grows. The largest eigenvalue of $\mathcal{L}_{00}^{+}$, however, is the inverse of the smallest in magnitude and non-zero eigenvalue of $\mathcal{L}_{00}$, which is also known as the spectral gap $\varepsilon$ of $\mathcal{L}_{00}$. If $\varepsilon$ scales as $\varepsilon = \Omega(n^{-\alpha})$, for some constant $\alpha > 0$, the largest eigenvalue of the Drazin inverse $\mathcal{L}_{00}^{+}$ grows at most with $n^\alpha$. Consequently, we can also estimate $\lambda_{ij} = O(n^{(i+j-1)\alpha})$ in the limit of large $n$, where the contribution from $(\mathcal{L}_{00}^{+})^{i+j-1}$ dominates. What we were originally interested in is whether the coefficients $\lambda_{1k}$ in eq. (D13) and $\lambda_{2k}$ in eq. (D14) can be bounded by a polynomial in $n$ whose exponent does not grow faster than linearly in $k$. Given the bounds for the $\lambda_{ij}$ we have just established under the assumption that the spectral gap closes only polynomially, $\varepsilon = \Omega(n^{-\alpha})$, we are guar-

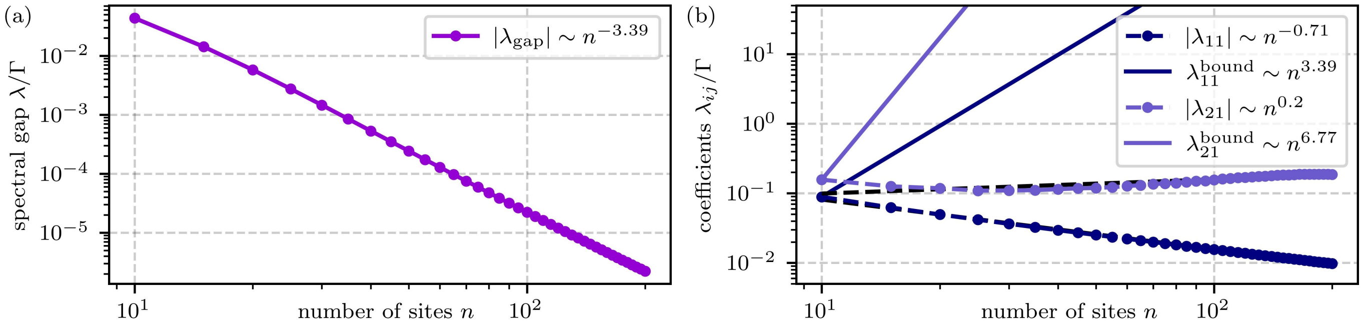


FIG. 6. Here we visualize the scaling of the spectral gap and the expansion coefficients of $\mathcal{L}$. In (a), we show how the spectral gap of the Liouvillian $\mathcal{L}$ scales as a function of the number of ring sites $n$. The spectral gap is the smallest magnitude non-zero eigenvalue of $\mathcal{L}$. We find that the gap closes only polynomially, which is ultimately the reason why it is sufficient to chose $\delta$ to decay polynomially in $n$ to make the perturbations to the clock precision vanish. (b) We show how the first two expansion coefficients of the dominant eigenvalue of $\mathcal{L}(\chi, \delta)$) scale as a function of $n$, to visualize the (loose) upper bounds obtained in (D19), using the spectral gap scaling.

anteed that

$$\lambda_{1k} = O(n^{\alpha k}) \text{ and } \lambda_{2k} = O(n^{(k+1)\alpha}). \tag{D19}$$

Figure 6(a) shows that it is indeed the case that the spectral gap only closes polynomially, at least for values of up to $n = 200$ that were numerically examined. The exponent found is $\alpha = 3.39$ (rounded to 2 digits). In Fig. 6(b), we see that the bounds in (D19) are satisfied, but loose. The underlying reason for the looseness is that in our estimate, we only accounted for the maximum eigenvalue of $\mathcal{L}_{00}^{+}$. However, the other terms in the series may shrink with $n$; for example, the steady-state populations encoded in $\omega$, become smaller as $n$ grows. For an upper bound, however, it is sufficient to consider the scaling of the largest values, without taking into account possible terms that improve the scaling in practice. Coming back to the prescription that $\delta = n^{-\zeta}$, it turns out as shown in Fig. 5(a) that a choice of $\zeta = 4$ is already sufficient to ensure the perturbations from eqs. (D13) and (D14) are negligible for the clock precision.

## Appendix E: Amplitude damping noise in the bulk

In this section, we provide additional detail how amplitude damping noise affecting the ring clock is modeled, providing additional detail for the simulation results from Extended Data Fig. 3 in the main text. Recalling from the Methods, such noise can lead to losses in the bulk of the ring. We have modeled this as a premature ticks with a subsequent re-initialization of the clock (a discussion of this model can be found at the end of this supplemental section). For example, for a loss from site $j = 1, \ldots, n-2$, this process is described by the jump operator

$$L_j = \sqrt{\Gamma \varepsilon_{\rm env}}\, |0\rangle\langle j|\,. \tag{E1}$$

The fraction $\varepsilon_{\rm env}$ describes the erroneous loss rate into the environment, expressed relative to the rate $\Gamma$ of the desired tick process $J = \sqrt{\Gamma}\, |0\rangle\langle n-1|$. For a finite-temperature environment, detailed balance requires that for any jump process, also its reverse must be present. In case of the tick process, this implies the reverse process of $J$, that is $J^\dagger$, has rate suppressed by a factor $e^{-\Sigma_{\rm tick}}$ as treated in detail in Sec. D. For losses in the bulk, a finite temperature environment gives rise to the following processes:

For one, we consider the reverse process of $L_j$ with rate suppressed by a factor $e^{-\Sigma_{\rm env}}$, where $\Sigma_{\rm env}$ is formally the entropy production in the environment to which the bulk couples. For another, and more relevant, is the possibility that the bulk of the ring accidentally absorbs an excitation from the environment. This process is described by a jump operator going from the single-excitation to the double-excitation subspace,

$$L_{(ji),j} = e^{-\Sigma_{\rm env}/2} \sqrt{\Gamma \varepsilon_{\rm env}}\, |j,i\rangle\langle j|\,, \tag{E2}$$

which we have already defined in eq. (14) of the Methods. We recall that this operator describes the process the ring clock initially having a single excitation on site $j = 0, 1, \ldots, n-1$ and then absorbing an excitation on a different site $i \neq j$. Also the emission process is possible, given by the operator

$$L_{j,(ji)} = \sqrt{\Gamma \varepsilon_{\rm env}}\, |j\rangle\langle j,i|\,, \tag{E3}$$

where the rate differs by a factor $e^{-\Sigma_{\rm env}}$ due to detailed balance. The exponent $\Sigma_{\rm env}$ is the entropy produced in the environment when an excitation is lost. For an environment at inverse temperature $\beta_{\rm env}$, the entropy production can be related to the dissipated heat $\Sigma_{\rm env} = \beta_{\rm env}\omega$, with $\omega$ the bare qubit frequency.

The nearest-neighbor hopping interactions between the different sites preserve the excitation numbers implying that the full Hamiltonian splits into separate contributions from each Fock-space. Hence, in the single-excitation subspace, the Hamiltonian is still given by the expression from eq. (2) in the main text. When it comes

to the double-excitation subspace, the Hamiltonian takes the form

$$H_2 = \sum_{j=0}^{n-3} \left[ \sum_{i=j+2}^{n-1} g_j \, |j+1, i\rangle\langle j, i| + \sum_{i=j+1}^{n-2} g_i \, |j, i+1\rangle\langle j, i| \right] + \text{h.c.}, \tag{E4}$$

with the couplings $g_j$ and $g_i$ the same as in the single-excitation case. For occupations on site $i$ and $j$, with $j < i$, the interactions in eq. (E4) generate the exchange of excitations between nearest neighbours $|j, i\rangle \to |j+1, i\rangle$ or $\to |j, i+1\rangle$ (including the Hermitian conjugate process) so long as the nearest neighbor is not yet occupied, i.e., $j+1 = i$.

In the double-excitation subspace, there is furthermore still the possibility of a usual tick taking place. For example, it may be that an excitation arrives at the last site $|n-1\rangle$ and ticks to the first site $|0\rangle$, while there is a second excitation somewhere different on site $j$. Extending the tick operator $J$ from the main text to the double-excitation subspace yields

$$J_2 = \sqrt{\Gamma} \sum_{j=1}^{n-2} |0, j\rangle\langle n-1, j| \, . \tag{E5}$$

The sum excludes the indices $j \neq 0, n-1$ because ticks can only occur when the first and last site are not occupied. The backward tick is generated by the reverse process, $\overline{J}_2 = e^{-\Sigma_{\text{tick}}/2} J_2^\dagger$ like in the single-excitation subspace case.

*Higher thermal occupations.* In principle, the bulk could also absorb further excitations, possibly leading to a further detrimental impact on the clock's precision. As we show in the following, however, this turns out not to be the case. The dominant errors due to interactions with the environment come from the lowest occupied subspace, i.e., the double-excitation subspace, and the contributions from the higher occupied subspaces are exponentially suppressed in the occupation number, so long as the environment is 'cold enough', in a sense which we clarify. For a bare qubit frequency $\omega$ and the environment's inverse temperature $\beta_{\text{env}}$, the thermal occupation probability of the subspace with $k \geq 1$ excitations is given by the Gibbs distribution [24],

$$p_k = \frac{1}{Z} \binom{n}{k} e^{-k\Sigma_{\text{env}}}, \tag{E6}$$

where $\Sigma_{\text{env}} = \beta_{\text{env}}\omega$ as before, and $\binom{n}{k}$ the binomial factor accounting for the number of states within the $k$-excitation subspace. The factor $Z$ is the partition function normalizing the probability distribution. The binomial factor can be bounded with $\binom{n}{k} \leq n^k/k!$ for all integer values of $k \leq n$, which allows us to bound (dropping pre-factor $Z$ that is constant for all $k$),

$$p_k \lesssim \frac{n^k}{k!} e^{-k\Sigma_{\text{env}}} = \frac{1}{k!} e^{-k(\Sigma_{\text{env}} - \log n)}. \tag{E7}$$

This reveals that if $\Sigma_{\text{env}} \geq \log n$, the thermal occupation probability of the $k$-excitation subspace super-exponentially decreases in $k$ (also note the $1/k!$ factor). For example, the choice $\Sigma_{\text{env}} = \frac{3}{2}\Sigma_{\text{tick}}$ would ensure such a scaling, also guaranteeing that the entropy due to the unwanted environmental interactions only scale logarithmically in the ring's number of sites like the tick entropy.

To a good approximation, and for sufficiently large $n$, modeling only the error due to the double-excitation subspace thus captures the dominant contributions from the environment's interactions. This allows for a computationally more efficient treatment of the errors because the first two excited subspaces $\mathcal{H}_1$ and $\mathcal{H}_2$ have dimensionality

$$\dim \mathcal{H}_1 \oplus \mathcal{H}_2 = \frac{n^2 + n}{2}, \tag{E8}$$

whereas the full Fock space with has dimension $2^n - 1$.

*Effective model.* Modeling the full 1-particle and 2-particle interactions to determine the clock precision $\mathcal{N}_\Sigma$ at finite entropy production $\Sigma_{\text{tick}}$ is computationally feasible for about $n \lesssim 12$. The reason is that the dimension (E8) grows quadratically in $n$ compared to the linear grows of the idealized model in the single-excitation subspace. To still verify the precision scaling up to $n = 200$ in presence of interactions with the environment, we can find an effective description with only a single state describing the impact of the full double-excitation subspace. Let us denote this effective state as $|\mathcal{H}_2\rangle$. The jumps $|j\rangle \to |j, i\rangle$ as defined by the operator in eq. (E2) can be effectively modeled by the jump operators

$$L_j^\uparrow = e^{-\Sigma_{\text{env}}/2} \sqrt{(n-1)\Gamma\varepsilon_{\text{env}}} \, |\mathcal{H}_2\rangle\langle j| \, . \tag{E9}$$

The $(n-1)$-fold increased rate comes from the $n-1$ possible sites on which the ring can absorb a second excitation given that it initially starts in $j$. The reverse process can then be effectively modeled as

$$L_j^\downarrow = \sqrt{2\Gamma\varepsilon_{\text{env}}} \, |j\rangle\langle \mathcal{H}_2| \, . \tag{E10}$$

Note that here, the rate is only increased by a factor 2 because when the system is in the doubly-occupied state $|j, i\rangle$ there are exactly two ways to decay: to $|j\rangle$ or $|i\rangle$.

While this effective model only captures simplified dissipative contributions from the amplitude-damping noise and forgoes the coherent interactions in the double-excitation subspace due to $H_2$, it turns out to well approximate the noise impact on the clock precision. We numerically verify the validity of the effective description as shown in the inset of Extended Data Fig. 3(a) from the main text. The numerically exact simulations show that the deviations between the true $(n^2 + n)/2$-dimensional model and the effective $(n+1)$-dimensional model decrease with the ring size. The comparison also confirms that some coherent effects in the double-excitation subspace are not captured by the effective model, resulting in different rates at which the deviation between true and

effective model decreases for even and odd numbers of sites $n$. Nonetheless, for both odd and even $n$, the deviations due to this simplification become negligible already for $n \sim 10$ sites. For ring lengths $n > 12$, as shown in the panel Extended Data Fig. 3(a), we thus calculate the clock precision $\mathcal{N}_\Sigma$ using the effective model, allowing us to simulate the noise for rings of up to $n = 200$ sites.

*Loss and re-initialization.* In the present error model, losses from the single-excitation subspace are modeled as spurious ticks, meaning the clock is prematurely re-initialized when a photon is lost in the bulk. This model accurately captures the back-action of the ring's bulk coupling to the environment, yet it relies on the ability to re-initialize the clock. Any realistic physical system inevitably has some non-zero failure rate, thus also the re-initialization may not succeed and the clock stops ticking with some small, but non-zero probability, say $p_{\text{fail}} > 0$. Akin to the threshold theorem in fault-tolerant quantum computing [25, 26], however, using multiple clocks redundantly can allow for exponentially suppressing the total failure probability while at most introducing a polynomial overhead in the thermodynamic cost. Using, $K$ clocks in parallel, the total probability of failure in each round is $\sim p_{\text{fail}}^K$, and exponentially vanishes in the number of copies. At the same time, the resources consumed only grow polynomially in $K$: A linear contribution is attributed to the additivity of the entropy. Another potentially polynomial contribution accounts for post-processing the output of the $K$ clocks. In summary, even if in a realistic scenario a total failure can never be eternally avoided, the above argument shows that such errors can be mitigated in a way that does not compromise the exponential entropy-precision relationship of the ring clock.


[1] G. Schaller, *Open Quantum Systems Far from Equilibrium* (Springer International Publishing, 2014).
[2] P. Erker, M. T. Mitchison, R. Silva, M. P. Woods, N. Brunner, and M. Huber, Autonomous Quantum Clocks: Does Thermodynamics Limit Our Ability to Measure Time?, Phys. Rev. X **7**, 031022 (2017).
[3] M. P. Woods, R. Silva, G. Pütz, S. Stupar, and R. Renner, Quantum Clocks are More Accurate Than Classical Ones, PRX Quantum **3**, 010319 (2022).
[4] A. P. T. Dost and M. P. Woods, Quantum advantages in timekeeping: dimensional advantage, entropic advantage and how to realise them via berry phases and ultra-regular spontaneous emission, 2303.10029 [quant-ph] (2023), pre-print.
[5] F. Meier, E. Schwarzhans, P. Erker, and M. Huber, Fundamental accuracy-resolution trade-off for timekeeping devices, Phys. Rev. Lett. **131**, 220201 (2023).
[6] A. J. Daley, Quantum trajectories and open many-body quantum systems, Advances in Physics **63**, 77 (2014).
[7] G. T. Landi, M. J. Kewming, M. T. Mitchison, and P. P. Potts, Current fluctuations in open quantum systems: Bridging the gap between quantum continuous measurements and full counting statistics, PRX Quantum **5**, 020201 (2024).
[8] P. Rouchon and A. Sarlette, Contraction and stability analysis of steady-states for open quantum systems described by Lindblad differential equations, in *52nd IEEE Conference on Decision and Control* (2013) pp. 6568–6573.
[9] F. Nicacio, M. Paternostro, and A. Ferraro, Determining stationary-state quantum properties directly from system-environment interactions, Phys. Rev. A **94**, 052129 (2016).
[10] S. L. Brunton and J. N. Kutz, *Data-Driven Science and Engineering: Machine Learning, Dynamical Systems, and Control* (Cambridge University Press, 2019).
[11] R. H. Bartels and G. W. Stewart, Algorithm 432 [C2]: Solution of the matrix equation $AX + XB = C$ [F4], Commun. ACM **15**, 820–826 (1972).
[12] E. Fradkin, *Field Theories of Condensed Matter Physics*, 2nd ed. (Cambridge University Press, 2013).
[13] R. Shankar, *Quantum Field Theory and Condensed Matter: An Introduction* (Cambridge University Press, 2017).
[14] L. Evans, *Partial Differential Equations* (American Mathematical Society, 2010).
[15] P. Chak and J. E. Sipe, Minimizing finite-size effects in artificial resonance tunneling structures, Opt. Lett. **31**, 2568 (2006).
[16] M. Sumetsky and B. J. Eggleton, Modeling and optimization of complex photonic resonant cavity circuits, Opt. Express **11**, 381 (2003).
[17] V. S. Ferreira, J. Banker, A. Sipahigil, M. H. Matheny, A. J. Keller, E. Kim, M. Mirhosseini, and O. Painter, Collapse and Revival of an Artificial Atom Coupled to a Structured Photonic Reservoir, Phys. Rev. X **11**, 041043 (2021).
[18] S. Datta, *Quantum Transport: Atom to Transistor* (Cambridge University Press, 2005).
[19] Y. V. Sokhotskii, *On definite integrals and functions used in series expansions*, Ph.D. thesis, St. Petersburg (1873).
[20] J. Plemelj, *Problems in the Sense of Riemann and Klein*, Interscience tracts in pure and applied mathematics (Interscience Publishers, 1964).
[21] L. D. Landau and E. M. Lifshitz, *Quantum Mechanics*, 2nd ed., Vol. 3 (Pergamon Press, 1977).
[22] D. R. Cox, *Renewal Theory* (Methuen & Co Ltd, 1962).
[23] R. Silva, N. Nurgalieva, and H. Wilming, Ticking clocks in quantum theory, arXiv:2306.01829 [quant-ph] (2023), pre-print.
[24] L. D. Landau and E. M. Lifshitz, *Statistical Physics*, 3rd ed. (Butterworth-Heinemann, Oxford, 1980).
[25] D. Aharonov and M. Ben-Or, Fault-Tolerant Quantum Computation with Constant Error, in *Proceedings of the Twenty-Ninth Annual ACM Symposium on Theory of Computing*, STOC '97 (Association for Computing Machinery, New York, NY, USA, 1997) p. 176–188.
[26] E. Knill, R. Laflamme, and W. H. Zurek, Resilient Quantum Computation, Science **279**, 342 (1998).
